# Factors impacting effective altruism: revisiting heuristics and biases in charity in a replication and extension registered report of Baron and Szymanska (2011)

**DOI:** 10.1098/rsos.250290

**Published:** 2025-05-21

**Authors:** Mannix Chan, Gilad Feldman

**Affiliations:** ^1^ Department of Psychology, The University of Hong Kong, Hong Kong

**Keywords:** effective altruism, heuristics, utilitarianism, donations, efficacy, charity, cognitive biases, registered report, replication

## Abstract

Individuals who donate to charity may be affected by various biases and donate inefficiently. In a replication and extension registered report with a US Amazon Mechanical Turk sample using CloudResearch (*N* = 1403), we replicated studies 1 to 4 in Baron & Szymanska (Baron & Szymanska 2011 In *The science of giving: experimental approaches to the study of charity* (eds DM Oppenheimer, CY Olivola), pp. 215–235 (doi:10.4324/9780203865972-24)) with extensions on reputation and overhead funding. We found support for the effects of a preference for lower perceived waste (*d* = 0.70, 95% CI [0.41, 0.99]), lower past costs (*d* = 0.59, 95% CI [0.16, 1.02]), for the ingroup (*d* = 0.52, 95% CI [0.47, 0.58]), for having some diversification between charities (*d* = 0.63, 95% CI [0.47, 0.78] for single projects; *d* = 1.18, 95% CI [1.00, 1.36] for several projects versus one) and against forced charity (*d* = 0.29, 95% CI [0.21, 0.37]; nominally replicated, but has caveats regarding validity); as at least four of our five hypotheses were found to replicate, we conclude this as being a successful replication. Extending the replication, we found support for an unexpected preference for anonymity on donation allocation (opposite to our predictions; *d* = 0.54, 95% CI [0.46, 0.61]), and support for a preference towards paid-for overhead costs on donation allocation (*d* = 0.60, 95% CI [0.52, 0.68]). We discuss the implications and validity of these findings. All materials, data and code were made available on: https://doi.org/10.17605/OSF.IO/BEP78. This registered report has been officially endorsed by Peer Community in Registered Reports: https://doi.org/10.24072/pci.rr.100775.

## 1. Background

There are many factors that influence a person’s decision to donate money to a charitable cause. Bekkers & Wiepking [1] identified eight separate mechanisms that drive charitable giving: awareness of need, solicitation, costs and benefits, altruism, reputation, psychological benefits, values and efficacy. Some or all of these mechanisms may drive a person’s choice to donate.

Baron & Szymanska [2] proposed that utilitarianism, which they defined as ‘the totality of good that comes about from a choice’, should be the objective standard from which to evaluate the efficiency of any given donation. The efficiency of a donation, therefore, is defined as the amount of good that the donation does per unit of money. They argued that charitable donations should be made aiming to maximize the amount of good done possible using the same amount of money. However, people might not donate according to these standards due to heuristics, mental shortcuts driven by cognitive constraints aiming to minimize the use of cognitive resources, which end up going counter to the intended goal. Baron & Szymanska [2] coined the term ‘non-utilitarian heuristics’ to describe these, and their research demonstrated five heuristics that result in biases, systematic deviations from the utilitarian model: (i) waste, (ii) average cost, (iii) diversification, (iv) nationalism, and (v) forced charity.

We report a replication and extension registered report of Baron & Szymanska [2] with the following goals. Our first goal was to conduct a close, independent and well-powered replication of the target article and the effects of various non-utilitarian heuristics that drive charitable donations. Our second goal was to extend the target article’s design by addressing several further heuristics not addressed in the target article. We hope to gain a better understanding of the different effects that result in suboptimal donations that are not aligned with maximizing the overall good.

We begin by introducing the various heuristics and biases covered in Baron & Szymanska [2], then discuss our motivation for the current replication study and the target’s hypotheses and study design, and conclude with our replication and extension design, needed adjustments and added extensions.

### 1.1. Waste/overhead effect

Efficacy is one of the eight mechanisms that drive people to donate to charity according to Bekkers & Wiepking [1]. However, it can be difficult to evaluate the amount of good a donation of a certain amount will do, whereas it is often easier to evaluate other simpler factors (evaluability bias [3]). One such factor that people seem to pay more attention to is the relative amount of money a charity spends on overhead. Baron & Szymanska [2] demonstrated that people appear biased against charities that have a higher overhead, even if those charities are actually more efficient in the good that they do when taking overhead costs into account. To test the impact of waste/overhead effect on charitable giving (hypothesis 1), participants in studies 1 through 3 were asked how much money they would be willing to allocate towards a charity that spent less on advertising or overhead compared with a charity that spent more, when both charities save the same number of people with the same amount of money. In all three studies, it was found that participants allocated more towards the charity that spent less money on advertising/overhead.

Recent follow-up research by Caviola *et al*. [4] showed further support for the idea that, when presented separately, people seem more willing to donate more to charities with a low overhead ratio regardless of cost-effectiveness, and that this effect disappears when presented together due to a higher evaluability.

### 1.2. Past costs effect

Baron & Szymanska [2] demonstrated that people are less willing to donate when presented with a charity’s past costs. To test the past costs effect on charitable giving (hypothesis 2), participants in studies 1 and 2 were asked how much they would allocate towards a charity that was previously less effective compared with a previously more effective charity, if both charities are equally effective right now. For both studies, it was found that participants allocated more towards the charity that was previously more effective.

They argued that the utilitarian approach to maximizing efficiency in charitable donations should be to evaluate the ‘marginal benefit per marginal dollar’; i.e. the extra benefit gained for a new contribution of a certain size. This means that people tend to be biased, for example, towards a charity that was cheaper to set up compared with one that took more money to set up, even if they could do the same or more good with a new donation of the same size, since they inaccurately take into account the previous efficiency of the charity in their evaluation, not just their current efficiency.

### 1.3. Diversification effect

The diversification effect is the phenomenon where people seek variety even when there is no reason to diversify [5]. In the context of charitable giving, this effect can manifest in the tendency towards giving to many charities over a single charity in pursuit of a perceived fairer distribution [6]. Baron & Szymanska [2] argued that this effect extends even to cases where many charities are, overall, less efficient than a single charity.

To test the diversification effect on charitable giving (hypothesis 3), participants in studies 2 and 4 were asked how much they would allocate to a charity that is more efficient compared with a charity that is less efficient. Several versions of this item were presented; overall, they found that participants allocated more than nothing towards the charity that was less efficient. Additionally, in study 4, they found differences between the allocation participants thought was right and what they thought was the most efficient allocation, both by the amount allocated and by the proportion of participants who allocated more than nothing to the less efficient charity. This means that they understood that the more efficient charity was indeed more efficient, yet still chose to donate more to the less efficient charity with the aim of diversifying their donations.

Using a different approach, they also asked participants in studies 1, 3 and 4 how much they would allocate towards a more efficient charity running one project and a less efficient charity running several. With this approach, participants were still found to allocate more than nothing towards the charity that was less efficient, but they found no differences between the allocation participants thought was right and what they thought was the most efficient allocation.

As a control condition, they also asked participants of studies 1 and 2 how much they would allocate towards a charity running one project and a charity running several if they were equally efficient; in this case, participants’ allocations were not found to differ from equal distribution.

### 1.4. Nationalism/ingroup effect

Another possible non-utilitarian heuristic that Baron & Szymanska [2] investigated is the effect of parochialism, which they defined as ‘a type of ingroup bias in which people weigh the welfare of their own group more heavily than those of outsiders’, commonly causing them to act in ways that benefit themselves over others. To test the ingroup effect on charitable giving (hypothesis 4), participants in studies 1 through 3 were asked how much they would allocate to a charity that helps children in their own country compared with a charity that helps children elsewhere. They presented several different versions of this item with the names of different locations inserted (e.g. India, Africa and Latin America). In all three studies, they found that participants allocated more money to the charity that helps children in their own country.

Baron & Szymanska [2] mainly framed this effect through the lens of nationalism; however, we can also look at the effect of parochialism as being a manifestation of the ingroup effect, where people tend to more positively evaluate a group to which they belong compared with an analogous group to which they do not [7].

Other studies have also shown that the ingroup effect appears in the context of charitable donations. For example, James & Zagefka [8] found that people were willing to donate approximately 30% more money when told that the victims of a flood were from their own country over that of a fictional one; in a study of real-life online crowdfunding, Burtch *et al*. [9] found that lenders preferred to lend money to those who were closer to them both in terms of culture and in physical distance.

### 1.5. Forced-charity/government-taxes effect

Baron & Szymanska [2] argued that people tend to prefer third-party organizations that collect voluntary donations for given beneficiaries over tax-supported government aid programmes. They labelled these systems of taxation as ‘forced charity’, and argued that if voluntary donations and taxation result in the same amount of benefit to the same group of people, a utilitarianism standard would mean that these two programmes should be evaluated equally, and that preferring one over the other would be a form of bias. To test the forced-charity effect on charitable giving (hypothesis 5), participants in study 4 were presented with a scenario where money needed to be raised, and were then asked to evaluate two different cases: one in which a tax covered the needed costs, and another where voluntary donations were used to cover the costs. Participants were asked to evaluate which case they preferred and thought was more fair. They presented several versions of this scenario in a random order. Findings were mixed; several versions showed support for a bias against forced charity, whereas some versions did not.

### 1.6. Choice of target for replication: Baron and Szymanska (2011)

We chose to replicate and extend Baron & Szymanska [2] based on several factors.

First, the article has had an impact on scholarly research and practice, especially in the field of effective altruism. At the time of writing this section (March 2023), this book chapter has received 109 citations according to Google Scholar, and has set the foundations for a new field on the psychology of (in)effective altruism. This new domain has led to some groundbreaking work by scholars like Caviola *et al*. [10] and Butts *et al*. [11], as well as other impactful studies in the field such as Burtch *et al*. [9] and Berman *et al*. [12], investigating charitable behaviour as affected by cultural differences and subjective preferences, respectively.

Second, the design of the studies in this paper allowed for the straightforward inclusion of extensions to allow for additional tests and insights on impediments to effective altruism. The formatting of the studies’ questionnaires lent itself well to the inclusion of selected extensions examining other factors that may preclude the effectiveness of participant choice in donation allocations, which we achieved by inserting items of a similar format to the original studies without heavily increasing the complexity of the replication, thus striking a balance between coverage of different impediments to effective altruism and the complexity of the study and the associated data analyses.

Finally, to the best of our knowledge, there are currently no published independent direct replications of this chapter, and there is much potential in revisiting and expanding on many of its insights. The target chapter was very brief on both the description of what was done, the analyses conducted and the results, and we hope that our reproduction of all the materials, the procedure, the analyses and the results produced would make it easier for others to follow and expand on this important work.

For these reasons, following the recent growing recognition of the importance of reproducibility and replicability in psychological science [13], we aimed to revisit Baron & Szymanska [2] by conducting a close, independent, and well-powered replication and extension registered report.

#### 1.6.1. Baron and Szymanska (2011): findings and hypotheses

Four studies were conducted by Baron & Szymanska in [2], and we aimed to replicate all four of them. The studies were originally conducted as online questionnaires; the original authors directed us to the original questionnaires’ availability online. We, therefore, only needed to make very minor adjustments to the study design, first by replicating the original questionnaire on Qualtrics, and second by adding extensions to the original questionnaire in the form of inserting additional items into two of the studies (to be discussed in the subsequent Extensions section).

We summarize the findings in the target article in table 1. Note that for ease of reading, this article (as well as the tables included within) follows the target article in that findings are sorted by hypothesis and not by study, as most hypotheses were tested over several different studies. Additionally, labels for each hypothesis follow that of the original study for ease of comparison.

**Table 1.** Summary of findings by Baron & Szymanska [2].

						95% confidence interval	
hypothesis	study	***p***	***t***	**d.f.**	Cohen’s ***d***	lower	upper
1 (waste)	1	= 0.0000	—	—	—	—	—
	2	—	—	—	—	—	—
	3	= 0.001	3.39	83	0.37	0.15	0.59
2 (average cost)	1	—^a^	—	—	—	—	—
	2	= 0.0005	3.66	76	0.42	0.19	0.64
3 (diversification)	1	—	—	—	—	—	—
	2	—	—	—	—	—	—
	3	—	—	—	—	—	—
	4 (means of responses)	= 0.0019	3.22	77	0.36	0.13	0.59
	4 (proportion of responses)	= 0.0006	3.61	77	0.41	0.18	0.64
4 (nationalism)	1	—	—	—	—	—	—
	2	—	—	—	—	—	—
	3	= 0.0000	—	—	—	—	—
5 (forced charity)	4 (version 1)	= 0.09	—	—	—	—	—
	4 (version 3)	= 0.0057	2.84	77	0.32	0.09	0.55
	4 (version 4)	= 0.0000	5.96	77	0.70	0.43	0.93

Versions of studies without a reported *p-*value are omitted for brevity.

Items marked with a dash (—) were not reported by the original authors or cannot be calculated.

We calculated the Cohen’s *d* and confidence interval values wherever possible; see the section on effect size calculations in the electronic supplementary material.

^a^ The result for this item was reported as significant by the original authors but the *p*-value was not mentioned.

### 1.7. Extensions

#### 1.7.1. Reputation/publicity effect

In the context of charitable giving, reputation refers to ‘the social consequences of donations for the donor’ [1]. As the act of donating money is usually seen as a positive thing to do, the act of being observed donating money to a charitable cause could lead to the positive consequence of one’s reputation increasing. A meta-analysis conducted by Bradley *et al*. [14] found that the feeling of being observed by others, whether actual or perceived, has a small but positive effect on prosocial behaviour. For example, in their study, Alpizar *et al*. [15] found that monetary donations made in public were 25% larger than ones made in private.

Therefore, we hypothesized that due to the additional benefits of having their reputation increase, people would show a preference towards donating to causes that could improve their reputation over ones that do not, even when efficiency is held constant.

#### 1.7.2. External funding effect

Hypothesis 1 of the replication states that participants are less willing to donate to charities with a higher perceived waste or overhead, even when efficiency is held constant. However, a study by Gneezy *et al*. [16] found that participants were more willing to donate to a charity with overhead costs if the costs were covered by another donor. Both the donation rate and the total amount of donations were found to increase when participants were told that the overhead costs are covered by another donor when compared to control groups and with other manipulations. Moreover, the findings in this paper were successfully replicated with an effect in the same direction in a mass replication effort by Camerer *et al*. [17].

Extending these findings, we hypothesized that when people are presented with both options (a charity where overhead is covered by another donor and a charity where overhead is paid for by the donor), people have a preference towards the charity in which overhead is covered by another donor, even if both charities are equally effective and spend the same amount on overhead.

We constructed the hypotheses of our replication from the results of the target article, and we summarize the hypotheses of our replication as well as our extensions in table 2.

**Table 2.** Summary of replication and extension hypotheses.

hypothesis	label	studies involved	description
replication
1	waste/overhead	1, 2, 3	people prefer to donate to charities with lower perceived waste, even when efficiency is held constant
2	past costs	1, 2	people prefer to donate to charities with lower past costs (higher average benefit per dollar), even when past costs are irrelevant in the context
3	diversification	1, 2, 3, 4	people tend to diversify their donations (donate to a larger number of charities), even when it means that their donations are less efficient overall
4	nationalism/ingroup	1, 2, 3	people prefer to donate to causes within their own country than to causes in other countries
5	forced charity/ government taxes	4	people prefer to help through voluntary donations over forced charity (government taxes)
extension			
6	reputation/publicity	1, 2	people prefer to donate publicly than to donate anonymously
7	external funding	1, 2	people prefer to donate to charities with overhead paid for by other donors

### 1.8. Pre-registration and open science

We provide all materials, data and code at: https://osf.io/bep78/. This registered report was submitted to *Royal Society Open Science* following peer review and recommendation for stage 2 acceptance at *Peer Community In* (PCI) *Registered Reports*’ platform. Full details of the peer review and recommendation of the paper at PCI Registered Reports may be found at the links below. After submission to the journal, the paper received no additional external peer review, but was accepted on the basis of the Editor’s recommendation according to our PCI registered reports’ policy (https://royalsocietypublishing.org/rsos/registered-reports#PCIRR). Stage 1 recommendation and review history: https://rr.peercommunityin.org/articles/rec?id=413; https://osf.io/gmswz/. Stage 2 recommendation and review history: Espinosa [18]; https://doi.org/10.24072/pci.rr.100775 [19]. All measures, manipulations and exclusions conducted for this investigation are reported, and data collection was completed before conducting the data analyses. The project was part of a large mass replication and extension project, which received ethics approval from the University of Hong Kong (no. EA220438). This registered report was written based on the registered report template by Feldman [20].

## 2. Method

### 2.1. Power and sensitivity analyses

The original article recruited about 80 participants per study, with some participants completing more than one study, for a total of approximately 273 participants overall (as clarified by the original authors). We conducted effect size calculations and power analyses based on the information and statistics reported by the target article. Based on the original article’s effect sizes, we found that the largest minimum sample size required in one study was 178 (for hypothesis 5: study 4, version 3). We ran the four studies separately, with participants evenly distributed, and therefore multiplied the minimum number of participants by four (178 × 4) resulting in 712.

However, to account for the possibility that the target’s effects were an overestimation, for possible exclusion of participants, and to allow for additional analyses, we conducted an analysis aiming for the ability to detect a Cohen’s *d* of 0.2 (power = 95%, alpha = 0.05) with one-sample and paired samples *t*-tests, commonly considered weak effects [21]. This required a sample size of 327 (and multiplied by 4 = 1308), for a larger total sample size of 1400 participants, accounting for possible exclusions due to incomplete data. We also note that this exceeds the 273 × 2.5 = 683 replication sample size as suggested by Simonsohn’s [22] rule of thumb of ‘replication sample size = 2.5 × original sample size’ (even if meant for other designs).

After we completed the data collection in stage 2, we noticed an oversight. We initially conducted our power analysis based on an alpha of 0.05, and based on the stage 1 peer review recommendation we adjusted our alpha to 0.005, yet we did not update our power analysis. This did not have much impact, as we targeted *d* = 0.2 which was an extreme underestimation. A sensitivity analysis of a sample size of 350 per study (1403/4), a power of 0.95 and an alpha of 0.005, one-sided, showed we were powered to detect a one-sample effect of *d* = 0.23, and a paired-sample effect of *d*_z_ = 0.23. We provide more information regarding these calculations in the section on ‘Analysis of the original article’ in the electronic supplementary material.

### 2.2. Participants

We recruited a total of 1403 US Amazon Mechanical Turk (MTurk) participants through CloudResearch [23] (*M*_age_ = 45.14, s.d. = 13.67; 687 males, 701 females, 15 other/did not disclose). We summarize a comparison of the target article sample and the replication sample in table 3.

**Table 3.** Comparison between the original study and replication participant demographics.

	Baron & Szymanska [2] (2011)	US MTurk workers on CloudResearch (2023)
sample size	approximately 273	1403
geographic origin	mostly Americans	US Americans
gender	About 80% female	687 male, 701 female, 15 other/did not disclose
median age (years)	about 42	42
average age (years)	not given	45.14
age s.d. (years)	not given	13.67
age range (years)	20−80	19−99
medium (location)	online questionnaire	online questionnaire
compensation	nominal payment	nominal payment
year	2011	2023

Based on our experience of running similar judgement and decision-making replications on MTurk, to ensure high-quality data collection, the following CloudResearch options were employed: Duplicate IP Block, Duplicate Geocode Block, Suspicious Geocode Block, Verify Worker Country Location, Enhanced Privacy, CloudResearch Approved Participants and Block Low Quality Participants.

Our initial assignment pay of 1.5 USD was based on the federal minimum wage of 7.25 USD an hour on a per minute basis. We first pretested survey duration with 30 participants to make sure our time run estimate was accurate and adjusted pay as needed. Due to the average completion time of the survey being longer than expected, pretest participants were each paid a bonus of 0.3 USD, making a total of 1.8 USD per participant; all other participants were paid 1.8 USD upon survey completion. Pretest participants’ responses were included in the final analysis.

### 2.3. Design and procedure: replication

We reconstructed the target’s stimuli and adapted it into an online Qualtrics survey based on the information provided in the article. Participants indicated their consent, with four questions confirming their eligibility, understanding and agreement with the terms of the study, to which they must answer with a ‘yes’ in order to proceed to the study. Three of the four questions also served as attention checks, with the option order being randomized per question (yes, no, not sure).

All participants completed only one of the four studies. At the end of the study, they answered a number of funnelling questions and provided their demographic information, two of which asked for the participant’s age and gender (*male/female/other/rather not disclose*), similar to the target article, and then were debriefed.

The complete list of items and questions considered in our analysis for both the replication and the extension sections in all four studies can be found in the ‘Materials used in the replication + extension’ section of the electronic supplementary material.

### 2.4. Manipulations

Participants saw the full set of items and questions in the study they were sorted into. Participants were first told to imagine that they have enough money for it to be easy for them to give some money away for charitable causes without seriously hurting their quality of life, and that they are willing to contribute some of their annual income to such causes. For each item, participants of each study were provided with descriptions of two different conditions, and asked to evaluate the two conditions using various scales; the order of these scales was not randomized, as the original studies did not do so. They were then given an optional open-ended feedback question—‘Any thoughts regarding this specific question? (optional; up to 255 characters)’— to discuss their thoughts about the presented item if they wished to (the responses to which do not factor into the quantitative analyses in both the replication and the extensions).

### 2.5. Measures

We provide a full list of the items used in this section in the electronic supplementary material and accompanying Qualtrics survey export files in the OSF folder.

### 2.6. Replication

#### 2.6.1. Waste/overhead

Participants of studies 1 through 3 read a description of two charities, which differed in the relative amount of money spent on advertising or overhead (e.g. study 1: ‘A and B help prevent deaths in children. Both of them can prevent five deaths for every $1000 of donations. A spends $200 out of every $1000 of donations on advertising. B spends $100’).

In studies 1 and 2, participants indicated the money allocation ratio between A and B (‘How much would you allocate to A/B?’) on an 11-point scale (0 = ‘A: 100%, B: 0%’; 100 = ‘A: 0%, B: 100%’; options at 10% intervals).

In study 3, participants were asked ‘What is the right allocation between A and B, ignoring your own feelings?’ (5-point scale; 1 = *all to* A; 2 = *more to* A*, some to* B; 3 = *equally to* A *and* B; 4 = *more to* B*, some to* A; 5 = *all to* B).

#### 2.6.2. Past costs

Participants in studies 1 and 2 read a description of two charities that differed in the overall cost per life already saved in the past, but that are equally efficient with new donations (e.g. study 2: ‘A and B will each prevent five deaths for every $10 000 of new donations. A was much more expensive to get started. Thus, the cost per life saved on average is higher for A, because A has spent more money in total’). They then indicated the money allocation ratio between A and B (‘How much would you allocate to A/B?’) on an 11-point scale (0 = ‘A: 100%, B: 0%’; 100 = ‘A: 0%, B: 100%’; options at 10% intervals).

#### 2.6.3. Diversification

##### 2.6.3.1. Unequal efficiency

For items in the ‘unequal efficiency’ condition, participants in studies 2 and 4 read several versions of a description of two charities that differed in their respective efficiency in saving lives (e.g. study 2, version 1: ‘A can save one life for $10 000. B can save one life for $12 500. The people helped are from the same groups, with the same problems’).

Participants in study 2 indicated the money allocation ratio to A and B (‘How much would you allocate to A/B?’) on an 11-point scale (0 = ‘A: 100%, B: 0%’; 100 = ‘A: 0%, B: 100%’; options at 10% intervals).

Participants in study 4 were asked the following questions: (i) *allocation*: ‘What is the right allocation between A and B, ignoring your own feelings?’, (ii) *feeling*: ‘What allocation would you feel best about making?’, (iii) *efficiency*: ‘What allocation between A and B would be the most efficient use of your money?’, and (iv) *impact:* ‘What allocation between A and B would do the most good for each $1000 spent?’ (all four questions were on a 5-point scale; 1 = a*ll to* A; 2 = *more to* A*, some to* B; 3 = *equally to* A *and* B; 4 = *more to* B*, some to* A; 5 = *all to* B).

##### 2.6.3.2. Unequal efficiency, several projects versus one

For the items in the ‘unequal efficiency, several projects versus one’ condition, participants in studies 1, 3 and 4 read a description of two charities that differed in that one charity is less efficient but helps more groups of people than the other (e.g. study 1: ‘A puts all the money into one project, which has a 75% chance of helping many children, and a 25% chance of doing no good at all. B puts the money into several different projects, each of which has a 70% chance of helping some children, but a 30% chance of doing no good’).

Participants in study 1 indicated the money allocation ratio between A and B (‘How much would you allocate to A/B?’) on an 11-point scale (0 = ‘A: 100%, B: 0%’; 100 = ‘A: 0%, B: 100%’; options at 10% intervals).

Participants in studies 3 and 4 were asked the following questions: (1) *allocation*: ‘What is the right allocation between A and B, ignoring your own feelings?’ (5-point scale; 1 = *all to* A; 2 = *more to* A*, some to* B; 3 = *equally to* A *and* B; 4 = *more to* B*, some to* A; 5 = *all to* B). Participants in study 4 were also asked about: (ii) *feeling:* ‘What allocation would you feel best about making?’, (iii) *efficiency:* ‘What allocation between A and B would be the most efficient use of your money?’, (iv) *impact:* ‘What allocation between A and B would do the most good for each $1000 spent?’ (all four questions were on a 5-point scale; 1 = *all to* A; 2 = *more to* A*, some to* B; 3 = *equally to* A *and* B; 4 = *more to* B*, some to* A; 5 = *all to* B). We note, though, that the target article only reported the findings regarding allocation and efficiency.

##### 2.6.3.3. Equal efficiency

In the control ‘equal efficiency’ condition, participants of studies 1 and 2 read a description of two charities that differed solely in the number of groups of children they helped (‘A puts all the money into one project, which will help 100 000 children. B puts the money into five different projects, each of which will help 20 000 children. (The benefit per child will be the same.)’). Participants then indicated the money allocation ratio between A and B (‘How much would you allocate to A/B?’) on an 11-point scale (0 = ‘A: 100%, B: 0%’; 100 = ‘A: 0%, B: 100%’; options at 10% intervals).

### 2.6.4. Nationalism/ingroup effect

Participants of studies 1 through 3 read one or several descriptions of two charities that differed in the groups they help; one helps children in their own country, and the other helps children around the world or in a specific foreign country/region (e.g. study 1: ‘A helps children who are in your own country. B helps children around the world. The children are equally needy’). Participants of studies 1 and 2 indicated the money allocation ratio between A and B (‘How much would you allocate to A/B?’) on an 11-point scale (0 = ‘A: 100%, B: 0%’; 100 = ‘A: 0%, B: 100%’ options at 10% intervals). Participants in study 3 indicated their perceived right allocation of money between A and B (‘What is the right allocation between A and B, ignoring your own feelings?’) on a 5-point scale (1 = *all to* A; 3 = *equally to* A *and* B; 5 = *all to* B).

### 2.6.5. Forced charity/government taxes: attitudes

Participants in study 4 read several scenarios about raising money. In each scenario, two cases were given: one where the money is raised through taxation (i.e. forced charity), and one where the money is raised by voluntary donations. For example, version 3 had the following:

Workers in your country who make widgets [imaginary goods] are getting lower wages because of competition from foreign imports. The price of widgets has gone down, and the workers have accepted wage cuts to avoid layoffs.

*Case A*: The government puts a tax on widgets. The proceeds from the tax are used to help the domestic workers by restoring their wages to their original level.

*Case B*: A voluntary charity collects funds to help the domestic workers. The funds are sufficient to restore their wages to their original level.

For each scenario, rather than the typical allocations in the other hypotheses, participants answered the following questions: (1) *preference:* ‘Which case would you favour if you had a choice?’ (−1 = *case A*; 0 = *both cases are equal*; 1 = *case B*), (ii) *fairness:* ‘Which case is more fair in distributing the cost and benefits?’, (iii) *freedom of choice*: ‘Which case provides more freedom of choice?’ (−1 = *case A*; 0 = *both cases are equal*; 1 = *case B*), and (iv) *importance:* ‘Which is more important in this scenario?’ (−1 = *fair cost allocation*; 0 = *both are equal*; 1 = *freedom of choice*). As in the target article, given high correlations between the items, they were averaged and compared against the midpoint of 0.

### 2.7. Replication: exploratory measures

When we reconstructed the survey based on the materials shared by the original authors, we realized that the authors included many additional measures in the design that were not reported in the book chapter. In our replication, we aimed to follow the methods of the original studies as closely as possible; we therefore chose to include these unreported measures in our studies as well. We provided all data for these unreported measures, and report a selection of analyses as additional exploratory measures, details of which can be found in the ‘Unused replication measures’ section in the electronic supplementary material.

### 2.8. Extensions

#### 2.8.1. Reputation/publicity

Participants in studies 1 and 2 read the description of two charities that differed only in that one publishes donor names and one does not (‘A and B both help thousands of children. A publishes the names of donors and how much they donated on their website. B keeps donors anonymous’). Participants indicated the money allocation ratio between A and B (‘How much would you allocate to A/B?’) on an 11-point scale (0 = ‘A: 100%, B: 0%’; 100 = ‘A: 0%, B: 100%’; options at 10% intervals).

#### 2.8.2. External funding

Participants in studies 1 and 2 read the description of two charities that differed only in that one charity used the donations to pay the overhead costs, whereas the other charity had another donor cover the overhead costs of the donation (‘A and B both help thousands of children. Both charities spend 50% of the donations they receive on administrative costs. For each $100 contribution to A, $50 will go to helping children and $50 will be used to cover administrative costs. For each $100 contribution to B, all $100 will go to helping children; another donor will cover the corresponding $100 administrative cost of this contribution’).^1^ Participants indicated the money allocation ratio between A and B (‘How much would you allocate to A/B?’) on an 11-point scale (0 = ‘A: 100%, B: 0%’; 100 = ‘A: 0%, B: 100%’; options at 10% intervals).

### 2.9. Evaluation criteria for replication findings

We aimed to compare our replication’s effects with those in the original article, wherever data were available, using the criteria set by LeBel *et al.* [24] (see the subsection ‘Replication evaluation’ in the electronic supplementary material).

We pre-registered our overall strategy to conclude a successful replication if at least 80% of the hypotheses/effects (i.e. 4 or 5 out of 5) showed a signal in the same direction as in the original study by Baron & Szymanska [2], a failed replication if only one or no studies (out of 5) showed a signal in the same direction as the original, and any mixed findings with lower than 80% and above 20% (i.e. 2 or 3 out of 5) to be a mixed results replication.

For each of the five hypotheses, there are multiple data sources from different studies, and the diversification hypothesis also has three sub-hypotheses, summarized in table 4. Each hypothesis was tested in 2−3 studies and some of the studies with multiple versions. We therefore calculated the (mini) meta-analytic effects for each of the hypotheses and concluded support for a hypothesis if the confidence intervals of the effect did not overlap with the null.

**Table 4.** Summary of replication statistical tests (one-sample *t*-tests).

									target article	
hypothesis	sudy (version/item)	*M*	s.d.	*t*‐test midpoint	*t*	d.f*.*	*p*	Cohen’s *d* and 95% CI	Cohen’s *d* and 95% CI	interpretation
1 (waste/ overhead)	1	75.42	29.62	50	16.0	348	<0.001	0.86 [0.73, 0.98]		
	2	72.94	27.37	50	15.8	356	<0.001	0.84 [0.72, 0.96]		
	3	3.40	0.97	3	7.71	346	<0.001	0.41 [0.30, 0.52]	0.37 [0.15, 0.56]	signal—consistent
**mini meta-effect**								**0.70 [0.41, 0.99]**		**supported**
2 (past costs)	1	70.46	25.38	50	15.1	348	<0.001	0.81 [0.68, 0.93]		
	2	59.50	25.49	50	7.04	356	<0.001	0.37 [0.27, 0.48]	0.42 [0.19, 0.64]	signal—consistent
**mini meta-effect**								**0.59 [0.16, 1.02]**		**supported**
3(a) (diversification effect) (unequal efficiency)	2 (version 1)	18.63	24.45	0	14.4	356	<0.001	0.76 [0.64, 0.88]		
	2 (version 2)	14.96	21.64	0	13.1	356	<0.001	0.69 [0.58, 0.81]		
	2 (version 3)	13.03	19.43	0	12.7	356	<0.001	0.67 [0.56, 0.78]		
	4 (right allocation) (version 1)	1.33	0.71	1	8.86	349	<0.001	0.47 [0.36, 0.58]		
	4 (feeling) (version 1)	1.32	0.71	1	8.54	349	<0.001	0.46 [0.35, 0.57]		
	4 (efficiency) (version 1)	1.34	0.74	1	8.61	349	<0.001	0.46 [0.35, 0.57]		
	4 (impact) (version 1)	1.33	0.74	1	8.41	349	<0.001	0.45 [0.34, 0.56]		
	4 (right allocation) (version 2)	1.77	1.06	1	13.5	349	<0.001	0.72 [0.60, 0.84]		
	4 (feeling) (version 2)	1.76	1.07	1	13.2	349	<0.001	0.71 [0.59, 0.82]		
	4 (efficiency) (version 2)	1.62	1.05	1	11.0	349	<0.001	0.59 [0.48, 0.70]		
	4 (impact) (version 2)	1.68	1.08	1	11.7	349	<0.001	0.63 [0.51, 0.74]		
	4 (right allocation) (version 3)	1.53	0.86	1	11.5	349	<0.001	0.62 [0.50, 0.73]		
	4 (feeling) (version 3)	1.52	0.82	1	11.8	349	<0.001	0.63 [0.52, 0.75]		
	4 (efficiency) (version 3)	1.44	0.83	1	9.88	349	<0.001	0.53 [0.42, 0.64]		
	4 (impact) (version 3)	1.45	0.88	1	9.57	349	<0.001	0.51 [0.40, 0.62]		
**mini meta-effect**								**0.63 [0.47, 0.78]**		**supported**
3(b) (diversification effect) (unequal efficiency, several projects versus one)	1	31.09	30.78	0	18.9	348	<0.001	1.01 [0.88, 1.14]		
	3	2.62	1.23	1	24.5	346	<0.001	1.32 [1.17, 1.46]		
	4 (right allocation)	2.61	1.25	1	24.0	349	<0.001	1.29 [1.14, 1.43]		
	4 (efficiency)	2.52	1.33	1	21.4	349	<0.001	1.14 [1.01, 1.28]		
**mini meta-effect**								**1.18 [1.01, 1.35]**		**supported**
3(c) (diversification effect) (equal efficiency)	1	46.50	26.09	50	−2.50	348	0.013	−0.13 [−0.24, −0.03]		
	2	48.54	25.22	50	−1.09	356	0.276	−0.06 [−0.16, 0.05]		
**mini meta-effect**								**−0.09 [−0.17, −0.02]**		**not supported^a^**
4 (ingroup effect)	1	38.57	23.71	50	−9.01	348	<0.001	−0.48 [−0.59, −0.37]		
	2 (around the world)	37.76	23.89	50	−9.68	356	<0.001	−0.51 [−0.62, −0.40]		
	2 (India)	35.04	23.57	50	−12.0	356	<0.001	−0.63 [−0.75, −0.52]		
	2 (Africa)	37.11	24.13	50	−10.1	356	<0.001	−0.53 [−0.64, −0.42]		
	2 (Latin America)	35.91	23.19	50	−11.5	356	<.001	−0.61 [−0.72, −0.49]		
	3 (India)	2.60	0.84	3	−8.80	346	<0.001	−0.47 [−0.58, −0.36]		
	3 (Eastern Europe)	2.55	0.84	3	−9.91	346	<0.001	−0.53 [−0.64, −0.42]		
	3 (China)	2.46	0.86	3	−11.6	346	<.001	−0.62 [−0.73, −0.51]		
	3 (Africa)	2.64	0.89	3	−7.54	346	<0.001	−0.40 [−0.51, −0.30]		
**mini meta-effect**								**−0.52 [−0.58, −0.47]**		**supported**
5 (forced-charity/ government-taxes effect)	4 (version 1)	0.14	0.63	0	4.13	349	<0.001	0.22 [0.11, 0.33]		
	4 (version 2)	0.11	0.66	0	3.02	349	<0.001	0.16 [0.06, 0.27]		
	4 (version 3)	0.14	0.65	0	3.91	349	<0.001	0.21 [0.10, 0.31]	0.32 [0.09, 0.55]	signal—inconsistent, smaller
	4 (version 4)	0.14	0.63	0	4.18	349	<0.001	0.22 [0.12, 0.33]	0.70 [0.43, 0.93]	signal— inconsistent, smaller
	4 (version 5)	0.38	0.59	0	12.0	349	<0.001	0.64 [0.53, 0.76]		
**mini meta-effect**								**0.29 [0.21, 0.37]**		**supported**

Outcome interpretations are based on LeBel *et al*. [24] where target article Cohen’s *d* and 95% CI are available; see the electronic supplementary material for details.

N/A: not reported at all in target, comparison interpretation not possible.

^a^ All constituent *p-*values were above the 0.005 alpha threshold for this sub-hypothesis.

### 2.10. Replication closeness evaluation

We provide details on the classification of the replications using the criteria by LeBel *et al*. [25] in table 5 (see the ‘replication closeness evaluation’ section in the electronic supplementary material). We summarize the replication as being a ‘very close replication’.

**Table 5.** Replication classification based on LeBel *et al*. [25].

design facet	replication	details of deviation
effect/hypothesis	same	
IV construct	same	
DV construct	same	
IV operationalization	same	
DV operationalization	same
population (e.g. age)	similar	participants were from the USA; the original study had no such restriction but indicated that the sample was ‘mostly Americans’
IV stimuli	same	
DV stimuli	same	
procedural details	similar	minor modifications of the formatting and wording to enhance clarity and comprehension; see the ‘comparisons and deviations’ section of the electronic supplementary material for more details
physical settings	similar	online
contextual variables	similar	participants were recruited online via CloudResearch instead of through a panel
replication classification	very close replication	

### 2.11. Data analysis strategy

We conducted data analyses for both the replication and extension sections using RStudio (v. 2023.06.1.524, Posit team [26]; running R v. 4.2.2, R Core Team [27]) with the packages ‘effectsize’ [28], ‘haven’ [29], ‘psych’ [30], ‘report’ [31], ‘reshape’ [32], ‘rmdformats’ [33], ‘rstatix’ [34], ‘statsExpressions’ [35] and ‘tidyverse’ [36], while graphs were generated using the packages ‘afex’ [37], ‘dplyr’ [38], ‘ggplot2’ [39], ‘ggstatsplot’ [40], ‘haven’ [29], ‘labelled’ [41], ‘PMCMRplus’ [42], ‘sjlabelled’ [43] and ‘reshape’ [32].

#### 2.11.1. Target alpha 0.005 and corrections

The tests for some of the hypotheses involve several analyses on similar dependent variables in the same study, such as having three analyses in study 2 to test hypothesis 3. Following our recommender Romain Espinosa’s suggestion to compensate for multiple analyses, we adjusted our target alpha to 0.005 for individual analyses throughout. We will report raw *p*-values. For ANOVAs, we will report Holm corrections for multiple analyses and will report both raw and corrected *p*-values, but our criteria for signal will use the corrected *p*-values against the 0.005 alpha threshold.

Additionally, we pre-registered to complement our null hypothesis significance testing (NHST) reporting with Bayesian analyses reporting using ggstatsplot in the case of support for the null, yet given that we rejected the null in all hypotheses, we do not report Bayesian analysis quantifying the null. Our replication success criteria followed the NHST signal and directionality per the LeBel *et al*. [24] criteria.

#### 2.11.2. Replication

Data analyses for the replication were conducted according to the information provided in the original article. For most hypotheses, a one-sample *t*‐test was used to compare participant responses with an equal allocation, or to a 100% allocation to the one charity that was objectively more efficient or effective according to the utilitarian standards as set by Baron & Szymanska [2]. Paired *t*-tests were used to compare participant responses to different items in the same scenario or group of scenarios.

Additionally, for some studies that had multiple versions of items pointing towards the same hypothesis, we conducted one-way repeated measures ANOVA tests to test for differences between the versions.

#### 2.11.3. Extensions

The data analyses for the extensions will follow the same structure of analogous items in the replication; one-sample *t*-tests will be used to compare participant responses with an equal allocation. As the extension items and questions posed to participants of both studies 1 and 2 are exactly the same in both extensions, unlike in the replication, the participant responses in both studies will be coalesced and analysed as one single dataset.

#### 2.11.4. Assumption checks

We aimed to follow the target article in their analyses. The data analyses in the original article were conducted using parametric one-sample and paired sample *t*-tests; these tests run under assumptions of normality and/or homogeneity of variance. We believe this is justified even if normality is violated given the complexities inherent in normality tests and rerunning analyses with non-parametric tests [44].

However, if we fail to find support for the hypotheses and the assumptions, we will examine the possibility of normality and/or homogeneity violations for the failed analyses, and make adjustments accordingly. In case we find a violation of normality, we will conduct exploratory complementary non-parametric tests of the same tests to verify the validity of the results without the normality and/or variance homogeneity assumptions (with Wilcoxon tests being used in replacement of one-sample and paired *t*-tests and the Kruskal–Wallis test in replacement of the one-way ANOVA test), and with stricter criteria using an alpha of 0.005 to account for the multiple analyses.

## 3. Results

### 3.1. Replication of original analyses

#### 3.1.1. Waste/overhead (hypothesis 1)

**Figure 1.**
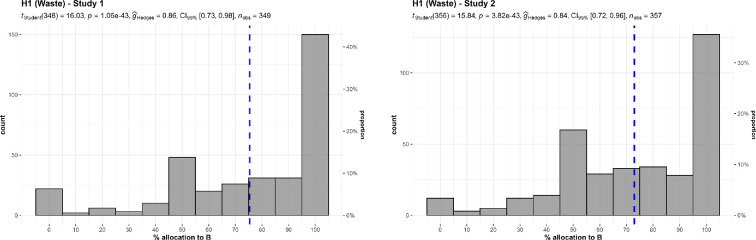
Waste/overhead: studies 1 and 2. Allocation between $200 advertising (A) and $100 advertising (B).The scenario: ‘A and B help prevent deaths in children. Both of them can prevent five deaths for every $1000 of donations (bolded only in the study 2 version). A spends $200 out of every $1000 of donations on advertising. B spends $100’.

**Figure 2.**
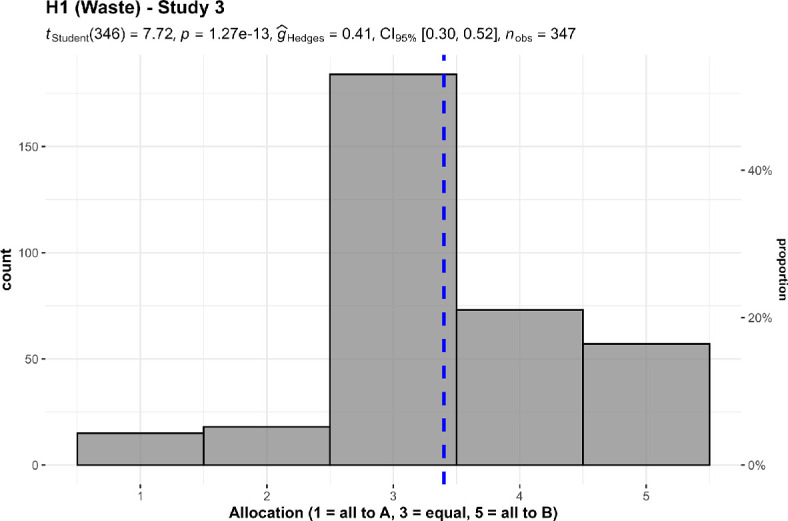
Waste/overhead: study 3. Allocation between $200 overhead (A) and $100 overhead (B).The scenario: ‘A and B help prevent deaths in children. Both of them can prevent five deaths for every $1000 of donations. A spends $200 out of every $1000 of donations on overhead expenses, but manages to save five lives with the remaining $800. B spends $100 out of every $1000 on overhead, and saves five lives with the remaining $900’.

We conducted three one-sample *t*-tests for studies 1 to 3, summarized and plotted in figures 1 and 2. The differences were that studies 1 and 2 were on a 0−100 scale and framed about advertising, whereas study 3 was on a 1−5 scale, framed about overhead, and is more explicit about efficiency with remaining funds.

We found support for the hypothesis that participants are less willing to donate to charities with a higher proportion of advertising (study 1: *M* = 75.42, s.d. = 29.62, *t*_348_ = 16.03, *p* < 0.001, *d* = 0.86, 95% CI [0.73, 0.98]; study 2 (*M* = 72.94, s.d. = 27.37, *t*_348_ = 15.84, *p* < 0.001, *d* = 0.84, 95% CI [0.72, 0.96]; both against a midpoint of 50), and with higher proportion of overhead (study 3: *M* = 3.40, s.d. = 0.97, *t*_346_ = 7.72, *p* < 0.001, *d* = 0.41, 95% CI [0.30, 0.52]; against a midpoint of 3).

#### 3.1.2. Past costs (hypothesis 2)

**Figure 3.**
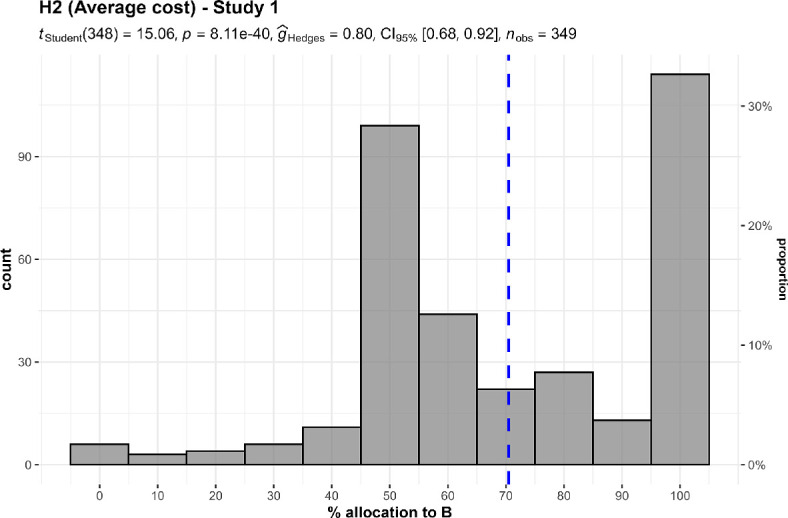
Past costs, study 1: allocation between two charities with similar current performance but with higher past costs (A) and lower past costs (B).The scenario: ‘A and B help prevent deaths in children. A prevents five deaths for every $1000 of donations, on average, and B prevents six deaths for every $1000. Given the donations they have received so far, and the opportunities for expansion, A will prevent five deaths for each *additional* $1000 beyond its current level of spending and B will also prevent five deaths’.

**Figure 4.**
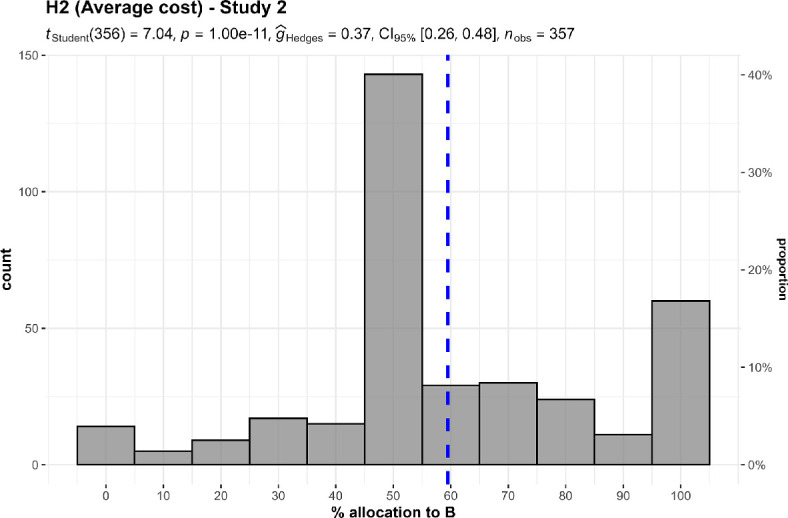
Past costs, study 2: allocation between two charities with similar performance but with higher past set-up costs (A) and lower past set-up costs (B).The scenario: ‘A and B will each prevent five deaths for every $10 000 of new donations. A was much more expensive to get started. Thus, the cost per life saved on average is higher for A, because A has spent more money in total’.

We conducted two one-sample *t*-tests for studies 1 and 2, which we summarize and plot in figures 3 and 4.

We found support for the hypothesis that when allocating between two charities with similar performance, participants allocated more money to a charity that had a record of lower past costs in the past (study 1: *M* = 70.46, s.d. = 25.38, *t*_348_ = 15.06, *p* < 0.001, *d* = 0.81, 95% CI [0.68, 0.93]; study 2: *M* = 59.5, s.d. = 25.49, *t*_348_ = 7.04, *p* < 0.001, *d* = 0.37, 95% CI [0.27, 0.48]; against a midpoint of 50).

#### 3.1.3. Diversification effect (hypothesis 3)

##### 3.1.3.1. Unequal efficiency

**Figure 5.**
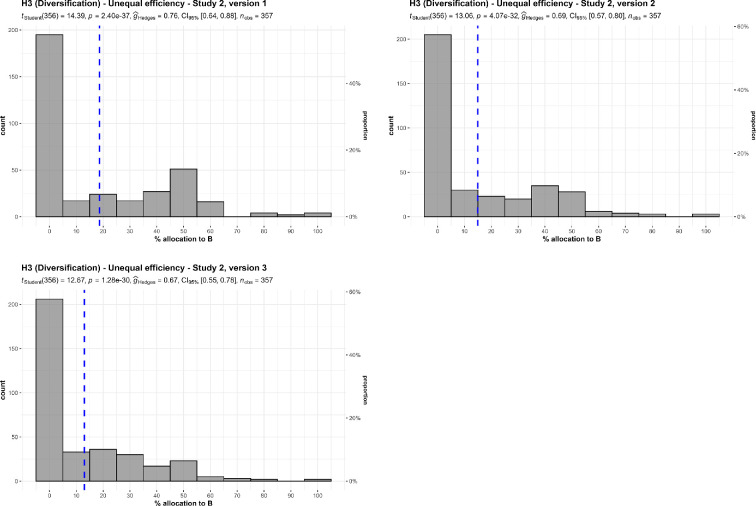
3a Diversification with unequal efficiency, study 2: allocation between a more efficient charity (A) and a less efficient charity (B). Scenarios: study 2, version 1: ‘A can save one life for $10 000. B can save one life for $12 500. The people helped are from the same groups, with the same problems’. Study 2, version 2: ‘A can save five lives for $50 000. B can save four lives for $50 000. The people helped are from the same groups, with the same problems’. Study 2, version 3: ‘A and B are both involved in preventing death in people with AIDS. A uses a method with a 75% chance of success over 5 years. B uses a method with a 50% chance of success over 5 years, with the same patients’

We conducted three one-sample *t*-tests for study 2, versions 1 to 3, which we summarize and plot in figure 5.

Unlike in the tests of the previous hypotheses, where both of the studies were equally efficient, in the empirical test of this hypothesis, charity A was clearly more efficient than charity B in the unequal efficiency scenarios, so the test we conducted was a one-sample *t*-test against 0, which allocates all funds to charity A.

We found support for the hypothesis that participants diversify their donations, even at the cost of inefficiency, meaning that on average participants did not allocate all funds to the charity that was clearly more efficient (version 1: *M* = 18.63, s.d.= 24.45, *t*_356_ = 14.40, *p* < 0.001, *d* = 0.76, 95% CI [0.64, 0.88]; version 2 (*M* = 14.96, s.d. = 21.64, *t*_356_ = 13.06, *p* < 0.001, *d* = 0.69, 95% CI [0.58, 0.81]; version 3: *M* = 13.03, s.d. = 19.43, *t*_356_ = 12.67, *p* < 0.001, *d* = 0.67, 95% CI [0.56, 0.78]; compared to the most efficient allocation, which is allocating 0% to the less efficient charity).

We also conducted twelve one-sample *t*-tests for versions 1, 2 and 3 in study 4. In all three versions, we found that not all participants allocated all funding to the more efficient charity (version 1: *M* = 1.33, s.d. = 0.71, *t*_349_ = 8.86, *p* < 0.001, *d* = 0.47, 95% CI [0.36, 0.58]; version 2: *M* = 1.77, s.d. = 1.06, *t*_349_ = 13.47, *p* < 0.001, *d* = 0.72, 95% CI [0.60, 0.84]; version 3: *M* = 1.53, s.d. = 0.86, *t*_349_ = 11.54, *p* < 0.001, *d* = 0.62, 95% CI [0.50, 0.73]), not all participants indicated that the more efficient charity would feel best (version 1: *M* = 1.32, s.d. = 0.71, *t*_349_ = 8.54, *p* < 0.001, *d* = 0.46, 95% CI [0.35, 0.57]; version 2: *M* = 1.76, s.d. = 1.07, *t*_349_ = 13.23, *p* < 0.001, *d* = 0.71, 95% CI [0.59, 0.82]; version 3: *M* = 1.52, s.d. = 0.82, *t*_349_ = 11.85, *p* < 0.001, *d* = 0.63, 95% CI [0.52, 0.75]), or that they perceived the more efficient charity to be the most efficient (version 1: *M* = 1.34, s.d. = 0.74, *t*_349_ = 8.61, *p* < 0.001, *d* = 0.46, 95% CI [0.35, 0.57]; version 2: *M* = 1.62, s.d. = 1.05, *t*_349_ = 11.05, *p* < 0.001, *d* = 0.59, 95% CI [0.48, 0.70]; version 3: *M* = 1.44, s.d. = 0.83, *t*_349_ = 9.88, *p* < 0.001, *d* = 0.53, 95% CI [0.42, 0.64]), or that it would do most good for each $1000 spent (version 1: *M* = 1.33, s.d. = 0.74, *t*_349_ = 8.41, *p* < 0.001, *d* = 0.45, 95% CI [0.34, 0.56]; version 2: *M* = 1.67, s.d. = 1.08, *t*_349_ = 11.07, *p* < 0.001, *d* = 0.63, 95% CI [0.51, 0.74]; version 3: *M* = 1.45, s.d. = 0.88, *t*_349_ = 9.57, *p* < 0.001, *d* = 0.51, 95% CI [0.40, 0.62]) (all compared against a lowest point of 1, with 1 being all to the more efficient charity and 5 being all to the less efficient charity).

**Figure 6.**
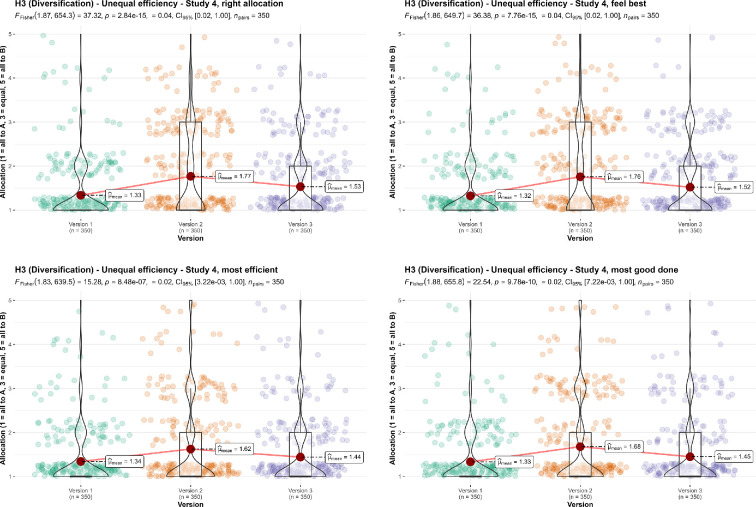
3a Diversification with unequal efficiency, study 4: allocation, feeling, efficiency and impact.Scale is ordinal; jitter was added for visualization. Scenarios: study 4, version 1: ‘ A and B are both involved in preventing death in people with AIDS. A uses a method with a 75% chance of success over 5 years. B uses a method with a 50% chance of success over 5 years, with the same patients’. Study 4, version 2: ‘A can save one life for $10 000. B can save one life for $12 500. The people helped are from the same groups, with the same problems’. Study 4, version 3: ‘A can save five lives for $50 000. B can save four lives for $50 000. The people helped are from the same groups, with the same problems’.

We then conducted four one-way repeated measures ANOVA tests, by collating the responses to versions 1, 2 and 3 in study 4, and found support for differences between the versions when asked about the *allocation* (*F*_1.87, 654.3_ = 37.32, *p* < 0.001, partial ω² = 0.04), *feeling* (*F*_1.86, 649.7_ = 36.38, *p* < 0.001, partial ω² = 0.04), *efficiency* (*F*_1.83, 639.5_ = 15.28, *p* < 0.001, partial ω² = 0.02), and *impact* (*F*_1.88, 655.8_ = 22.54, *p* < 0.001, partial ω² = 0.02). We summarize and plot all analyses in figure 6.

**Figure 7.**
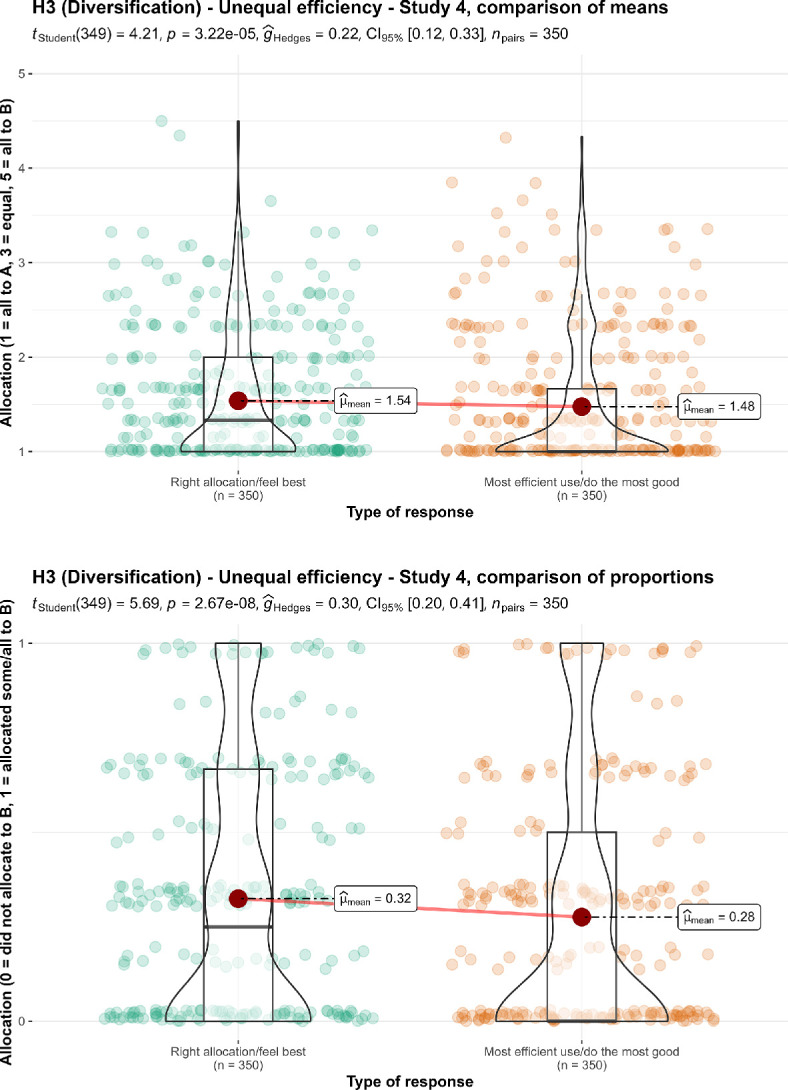
3a Diversification in unequal efficiency, study 4: comparison of allocation.Scale is ordinal; jitter was added for visualization purposes.

We then conducted a paired *t*‐test, and found support for differences between the average response per participant to the ‘right allocation’ and ‘allocation that feels best’ questions combined (*M* = 1.54, s.d. = 0.68) and the average response per participant to the ‘most efficient allocation’ and ‘allocation that does the most good’ questions combined (*M* = 1.48, s.d. = 0.69; *t*_349_ = 4.21, *p* < 0.001, *d* = 0.23, 95% CI [0.12, 0.33]). We also found support for differences between the proportion of responses that allocated something to the less efficient charity when asked for ‘right allocation’ and the ‘allocation that feels best’ (*M* = 0.32, s.d. = 0.36) to the proportion of responses that allocated something to the less efficient charity when asked for the ‘most efficient allocation’ and the ‘allocation that does the most good’ (*M* = 0.28, s.d. = 0.36; *t*_349_ = 5.69, *p* < 0.001, *d* = 0.30, 95% CI [0.20, 0.41]). We summarize and plot both analyses in figure 7.

##### 3.1.3.2. Unequal efficiency, several projects versus one

**Figure 8.**
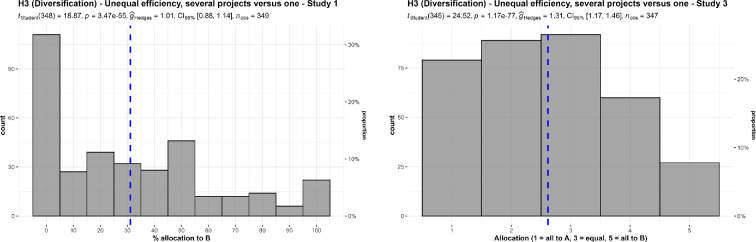
3b Diversification in unequal efficiency, studies 1 and 3: allocation between a single project more efficient charity (A) and a multiple project less efficient charity (B).Scenarios: study 1: ‘A puts all the money into one project, which has a 75% chance of helping many children, and a 25% chance of doing no good at all. B puts the money into several different projects, each of which has a 70% chance of helping some children, but a 30% chance of doing no good’. Study 3: ‘A puts $1 000 000 into one project, which has a 75% chance of helping 10 000 children, and a 25% chance of doing no good. B puts $200 000 into each of five projects ($1 000 000 total). Each of the five has a 70% chance of helping 2000 children and a 30% chance of doing no good. (If all five succeed, then the total benefit is for 10 000 children, the same as A.)’.

We conducted two one-sample *t*-tests for studies 1 and 3, which we summarize and plot in figure 8. We found support for the hypothesis that, on average, people would choose to diversify by allocating some funds to a multiple-project charity and do not allocate all funding to a more efficient single project charity, in study 1 (against a lowest point of 0; *M* = 31.09, s.d. = 30.78, *t*_348_ = 18.87, *p* < 0.001, *d* = 1.01, 95% CI [0.88, 1.14]) and in study 3 (against a lowest point of 1; *M* = 2.62, s.d. = 1.23, *t*_348_ = 24.52, *p* < 0.001, *d* = 1.32, 95% CI [1.17, 1.46]).

We also conducted two one-sample *t*-tests for study 4. We found support for higher than nothing allocation to the less efficient charity when asked for the right allocation (*M* = 2.61, s.d. = 1.25, *t*_349_ = 24.08, *p* < 0.001, *d* = 1.29, 95% CI [1.14, 1.43]) and the most efficient allocation (*M* = 2.52, s.d. = 1.33, *t*_349_ = 21.38, *p* < 0.001, *d* = 1.14, 95% CI [1.01, 1.28]) (both against a lowest point of 1, with 1 being all to the more efficient charity and 5 being all to the less efficient charity).

**Figure 9.**
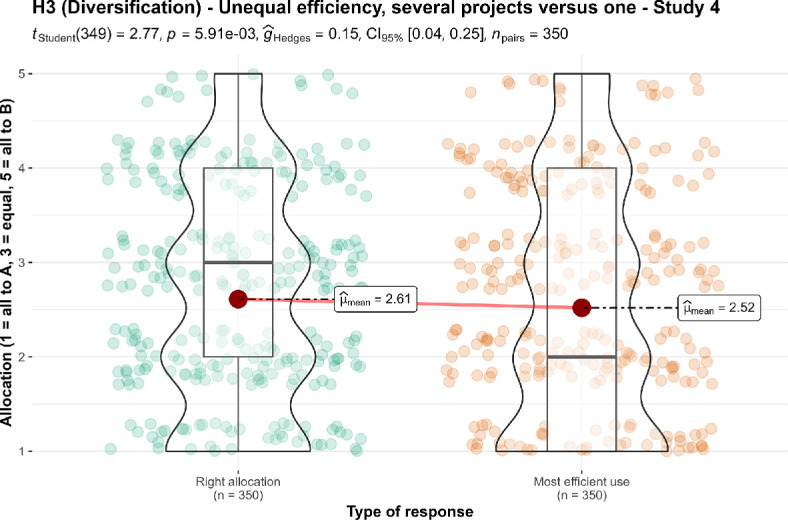
Diversification in unequal efficiency, study 4: comparison of allocation (several projects versus one).Scale is ordinal; jitter was added for visualization purposes. The scenario: ‘A puts $1 000 000 into one project, which has a 75% chance of helping 10 000 children, and a 25% chance of doing no good. B puts $200 000 into each of the five projects ($1 000 000 total). Each of the five has a 70% chance of helping 2000 children and a 30% chance of doing no good. (If all five succeed, then the total benefit is for 10 000 children, the same as A.)’.

We conducted a paired *t*‐test and found no support for differences between the responses to the two questions (*t*_349_ = 2.77, *p* = 0.006, *d* = 0.15, 95% CI [0.04, 0.25]; summarized and plotted in figure 9).

##### 3.1.3.3. Equal efficiency

**Figure 10.**
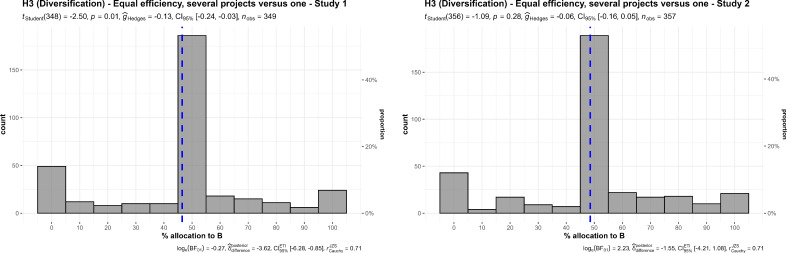
Diversification with equal efficiency, studies 1 and 2: allocation.The scenario: ‘A puts all the money into one project, which will help 100 000 children. B puts the money into five different projects, each of which will help 20 000 children. (The benefit per child will be the same.)’.

We conducted two one-sample *t*-tests for studies 1 and 2, summarized and plotted in figure 10. We found no support for preference towards either a single project charity or a multiple project charity in allocation, in study 1 (*M* = 46.50, s.d. = 26.09, *t*_349_ = −2.50, *p* = 0.013, *d* = −0.13, 95% CI [−0.24, −0.03]) and study 2 (*M* = 48.54, s.d. = 25.22, *t*_349_ = −1.09, *p* = 0.276, *d* = −0.06, 95% CI [−0.16, 0.05]), compared with an equal allocation of 50% each.

### 3.1.4. Nationalism/ingroup effect (hypothesis 4)

**Figure 11.**
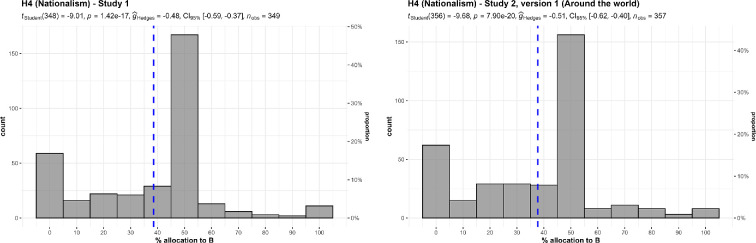
Ingroup effect, studies 1 and 2: allocation between charity in own country (A) and a global charity (B).Scenarios: study 1: ‘A helps children who are in your own country. B helps children around the world. The children are equally needy’. Study 2, version 1: ‘A helps children who are in your own country. B helps children around the world. The children are equally needy, and the benefits are the same for each child’.

We conducted two one-sample *t*-tests for studies 1 and 2, summarized and plotted in figure 11. We found support for a preference for allocating more money towards charities that help children in their own country over children around the world (study 1: *M* = 38.57, s.d. = 23.71, *t*_348_ = −9.01, *p* < 0.001, *d* = −0.48, 95% CI [−0.59, −0.37]; study 2: *M* = 37.76, s.d. = 23.89, *t*_356_ = −9.68, *p* < 0.001, *d* = −0.51, 95% CI [−0.62, −0.40]; both against a 50% midpoint).

We conducted three additional one-sample *t*-tests for study 2 against specific locations, and found support for the effect when comparing children in own country to children in India (*M* = 35.04, s.d. = 23.57, *t*_356_ = −11.99, *p* < 0.001, *d* = −0.63, 95% CI [−0.75, −0.52]), Africa (*M* = 37.11, s.d. = 24.13, *t*_356_ = −10.09, *p* < 0.001, *d* = −0.53, 95% CI [−0.64, −0.42]) and Latin America (*M* = 35.91, s.d. = 23.19, *t*_356_ = −11.48, *p* < 0.001, *d* = −0.61, 95% CI [−0.72, −0.49]) (all against a 50% midpoint).

**Figure 12.**
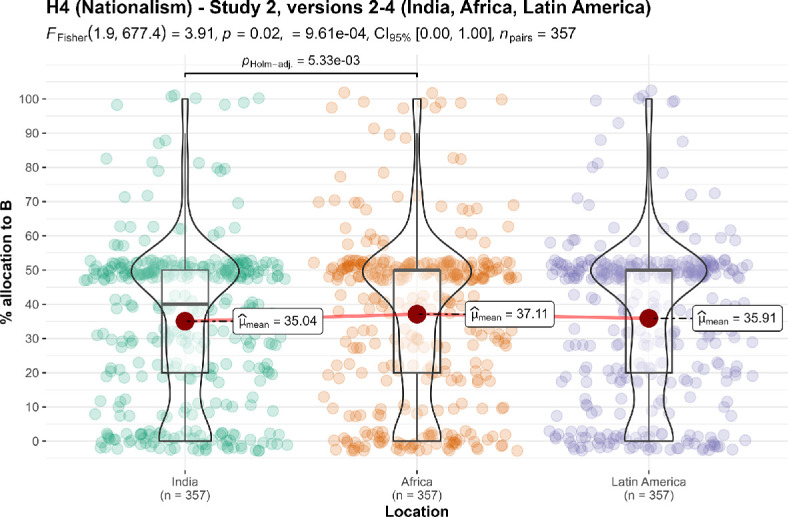
Ingroup effect in study 2, versions 2−4: comparison of allocation.Scale is ordinal; jitter was added for visualization purposes. Scenarios: study 2: ‘A helps children who are in your own country. B helps children in (version 2: India; version 3: Africa; version 4: Latin America). The children are equally needy, and the benefits are the same for each child’.

We then conducted a one-way repeated measures ANOVA test and found no support for differences between the three location conditions (*F*_1.90, 677.4_ = 3.91, *p* = 0.022, partial ω² = 0.00; summarized and plotted in figure 12).

**Figure 13.**
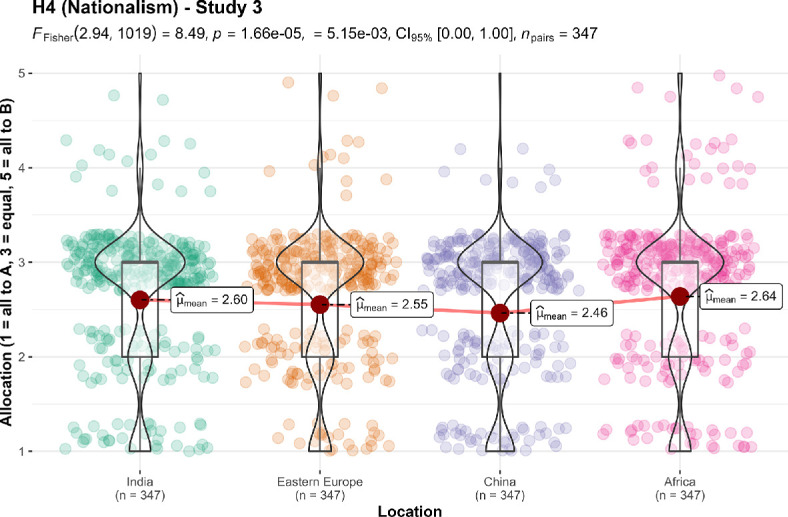
Ingroup effect in study 3, versions 1−4: comparison of allocation.Scale is ordinal; jitter was added for visualization purposes. Scenario: study 3: ‘A cures a disease in children who are in a distant part of your own country. B cures the same disease in children in (version 1: India; version 2: Eastern Europe; version 3: China; version 4: Africa). A and B are equally efficient. You do not know any of the affected children, or any children who have had this disease’.

We conducted four one-sample *t*-tests for study 3 against specific locations, and found support for the effect when comparing children in own country to children in India (*M* = 2.60, s.d. = 0.84, *t*_346_ = −8.80, *p* < 0.001, *d* = −0.47, 95% CI [−0.58, −0.36]), Eastern Europe (*M* = 2.55, s.d. = 0.84, *t*_346_ = −9.91, *p* < 0.001, *d* = −0.53, 95% CI [−0.64, −0.42]), China (*M* = 2.46, s.d. = 0.86, *t*_346_ = −11.56, *p* < 0.001, *d* = −0.62, 95% CI [−0.73, −0.51]) and Africa (*M* = 2.64, s.d. = 0.89, *t*_346_ = −7.54, *p* < 0.001, *d* = −0.40, 95% CI [−0.51, −0.30]) (all against a midpoint of 3, summarized and plotted in figure 13).

We also conducted a one-way repeated measures ANOVA test comparing the four conditions and found support for differences between the four location conditions (*F*_2.94, 1019_ = 8.49, *p* < 0.001, partial ω² = 0.00).

### 3.1.5. Forced-charity/government-taxes effect (hypothesis 5)

We conducted three one-sample *t*-tests for study 4, versions 1 through 5 using the average of the four questions asked per version, which we summarize and plot in figure S1 of the electronic supplementary material (in the section ‘Results: replication’).

We found support for the hypothesis that participants have a preference against forced charity in version 1 (*M* = 0.14, s.d. = 0.63, *t*_349_ = 4.13, *p* < 0.001, *d* = 0.22, 95% CI [0.11, 0.33]), version 2 (*M* = 0.11, s.d. = 0.66, *t*_349_ = 3.02, *p* = 0.003, *d* = 0.16, 95% CI [0.06, 0.27]), version 3 (*M* = 0.14, s.d. = 0.65, *t*_349_ = 3.91, *p* < 0.001, *d* = 0.21, 95% CI [0.10, 0.31]), version 4 (*M* = 0.14, s.d. = 0.63, *t*_349_ = 4.18, *p* < 0.001, *d* = 0.22, 95% CI [0.12, 0.33]) and version 5 (*M* = 0.38, s.d. = 0.59, *t*_349_ = 12.02, *p* < 0.001, *d* = 0.64, 95% CI [0.53, 0.76]) (all against an equal response of 0).

**Figure 14.**
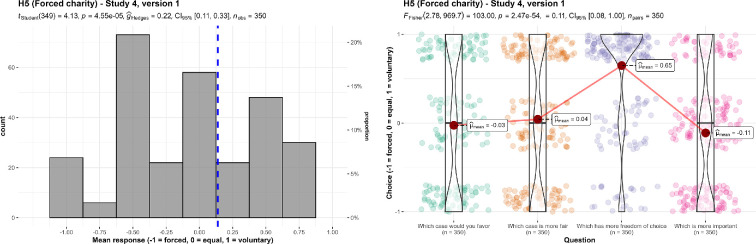
Forced charity in study 4, version 1: mean response and comparison of responses.Results in the first figure are the mean of the four dependent measures in the second figure: ‘which case would you favour’, ‘which case is more fair’, ‘which has more freedom of choice’ (−1 = the forced case; 0 = both cases are equal; 1 = the voluntary case), and ‘which (of fair cost allocation and freedom of choice) is more important’ (−1 = fair cost allocation; 0 = both are equal; 1 = freedom of choice). Scales are ordinal. Jitter was added for visualization purposes in the second figure.Scenario: version 1: ‘Your country requires everyone to buy health insurance. The fee is fixed at about $2500 per person. Case A: To help those who have trouble paying, the government levies a *special income* tax. Families earning less than $7500 per person pay no tax. Others pay a fixed percentage of their income above $7500/person. Case B: To help those who have trouble paying, charities collect *voluntary donations*. The charities distribute the funds to try to come as close as possible to the situation described in case A. That is, they provide a subsidy for families earning less than $7500/person, they reduce the subsidy gradually as income increases, and they solicit contributions from rich people who would pay less in case B than in case A. Suppose that the charities succeed, so that the bottom line is the same for each family as in case A’.

We then conducted five one-way repeated measures ANOVA tests and found support for differences in responses between the questions in version 1 (*F*_2.78, 969.7_ = 103.00, *p* < 0.001, partial ω² = 0.11), version 2 (*F*_2.73, 952.0_ = 91.77, *p* < 0.001, partial ω² = 0.10), version 3 (*F*_2.65, 923.4_ = 96.29, *p* < 0.001, partial ω² = 0.10), version 4 (*F*_2.80, 978.5_ = 57.48, *p* < 0.001, partial ω² = 0.06), and version 5 (*F*_(2.88, 1005)_ = 104.14, *p* < 0.001, partial ω² = 0.11). We summarize and plot all analyses in electronic supplementary material, figure S2 (in the section ‘Results: replication’); a representative example (version 1) is provided in figure 14 for easier reference.

### 3.2. Comparing replication to original findings

We provide a summary of replication statistical tests in tables 4 and 6. For tests corresponding to ones in the original study where enough details were provided to calculate an effect size, we interpreted the results of our replication based on the criteria in LeBel *et al.* [24] by comparing our replication effect sizes and confidence intervals to the original effect sizes in the target article.

**Table 6.** Summary of replication statistical tests (paired *t*-tests).

		condition 1			condition 2							target article	
hypothesis and study	comparison	label	*M*	s.d.	label	*M*	s.d*.*	*t*	d.f.	*p*	Cohen’s *d* and 95% CI	Cohen’s *d* and 95% CI	interpretation
3 (diversification effect), study 4 (unequal efficiency)	comparison of means	right allocation/feel best	1.54	0.68	most efficient use/most good done	1.48	0.69	4.21	349	<0.001	0.23 [0.12, 0.33]	0.36 [0.13, 0.59]	signal—inconsistent, smaller
	comparison of proportions	right allocation/feel best	0.32	0.36	most efficient use/most good done	0.28	0.36	5.69	349	<0.001	0.30 [0.20, 0.41]	0.41 [0.18, 0.64]	signal—consistent
(unequal efficiency, several projects versus one)	comparison of allocation	right allocation	2.61	1.25	most efficient use	2.52	1.33	2.77	349	0.006	0.15 [0.04, 0.25]		

Outcome interpretations are based on LeBel *et al*. [24] where target article Cohen’s *d* and 95% CI are available.

### 3.3. Extension analyses

We provide a summary of the extension findings in table 7.

**Table 7.** Summary of extension statistical tests (one-way *t*-tests)*.*

	*M*	s.d.	midpoint	*t*-stat	d.f.	*p*	Cohen’s *d* and 95% CI	conclusion
reputation (hypothesis 6)								
studies 1 and 2	65.27	28.47	50	14.25	705	<0.001	0.54 [0.46, 0.61]	support for anonymity; opposite effect
overhead funding (hypothesis 7)								
studies 1 and 2	66.57	27.51	50	16.01	705	<0.001	0.60 [0.52, 0.68]	supported

#### 3.3.1. Reputation/publicity (hypothesis 6)

**Figure 15.**
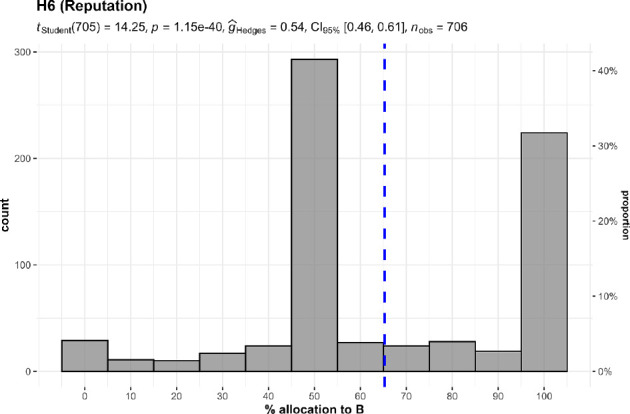
Reputation in studies 1 and 2 combined: allocation.Scenario: study 1, study 2: ‘A and B both help thousands of children. A publishes the names of donors and how much they donated on their website. B keeps donors anonymous’*.*

We conducted a one-sample *t*‐test and failed to find support for participant preference in donating to causes that could improve their reputation; we instead found support for participant preference in donating to causes that would keep them anonymous (against a 50% midpoint; *M* = 65.27, s.d. = 28.47, *t*_705_ = 14.25, *p* < 0.001, *d* = 0.54, 95% CI [0.46, 0.61]; summarized and plotted in figure 15).

#### 3.3.2. External funding (hypothesis 7)

**Figure 16.**
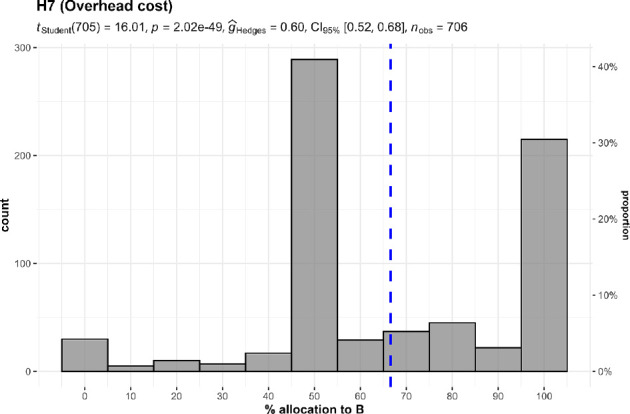
External funding in studies 1 and 2 combined: allocation.Scenario: study 1, study 2: ‘A and B both help thousands of children. Both charities spend 50% of the donations they receive on administrative costs. For each $100 contribution to A, $50 will go to helping children and $50 will be used to cover administrative costs. For each $100 contribution to B, all $100 will go to helping children; another donor will cover the corresponding $100 administrative cost of this contribution’.

We conducted a one-sample *t*‐test and found support for participant preference in donating to causes for which overhead has been paid for by another donor (against a 50% midpoint; *M* = 66.57, s.d. = 27.51, *t*_705_ = 16.01, *p* < 0.001, *d* = 0.60, 95% CI [0.52, 0.68]; summarized and plotted in figure 16).

### 3.4. *Post hoc* exploratory analyses

#### 3.4.1. Analysis of variance *post hoc* tests

We conducted *post hoc* comparisons for all ANOVA tests where we found support for differences between groups. Further details and results for these *post hoc* tests can be found in the ‘Additional analyses and results’ section of the electronic supplementary material.

#### 3.4.2. Re-run of hypothesis 5 with ‘freedom’ question excluded

We conducted five one-way repeated measures ANOVA tests for all five versions of hypothesis 5 and found support for differences in responses between the four questions in all five versions. *Post hoc* comparisons found support for differences in responses between the ‘Which has more freedom of choice’ question and each of the other three questions for all five versions (see the ‘Additional analyses and results—hypothesis 5’ section of the electronic supplementary material for more details). We therefore re-ran the data analyses for all five versions of hypothesis 5, but this time with the ‘Which has more freedom of choice’ question excluded.

We provide a summary of these statistical tests in table 8.

**Table 8.** Summary of post hoc exploratory statistical tests (one-sample *t*-tests).

hypothesis	study (version)	*M*	s.d.	*t*‐test midpoint	*t*	d.f.	*p*	Cohen’s *d* and 95% CI
5 (**excluding the ‘more freedom’ question**)	4 (version 1)	−0.03	0.75	0	−0.79	349	0.431	−0.04 [−0.15, 0.06]
	4 (version 2)	−0.05	0.76	0	−1.21	349	0.226	−0.06 [−0.17, 0.04]
	4 (version 3)	−0.02	0.76	0	−0.49	349	0.625	−0.03 [−0.13, 0.08]
	4 (version 4)	0.02	0.70	0	0.51	349	0.613	0.03 [−0.08, 0.13]
	4 (version 5)	0.23	0.72	0	6.04	349	<0.001	0.32 [0.21, 0.43]

## 4. Discussion

We conducted a very close replication and extension of four studies reported in Baron & Szymanska [2]. Our replication results were consistent with the original findings; we found support for each of our replication and extension hypotheses, though we suggest caution with interpreting the findings of hypothesis 5 (forced charity/government taxes; further elaborated below in the ‘Limitations’ section).

### 4.1. Replication

For hypotheses 1 and 2, our replication results were highly consistent with those of the original study for all of the waste and the past cost conditions, showing that with efficiency kept constant (number of lives saved), people have a clear preference towards charities with lower perceived waste (advertising or overhead) and lower past costs (previous performance record or set-up costs), respectively. The effects we found for these two hypotheses were very consistent with the ones provided in the target article, given the change in time, population and sample size. For hypothesis 1 in particular, the difference between the effect sizes found in studies 1 and 2 (*d* = 0.86 and 0.84, respectively) and study 3 (*d* = 0.41) may be due to a difference in the type of waste: ‘advertising’ might have had a stronger connotation of waste compared with ‘overhead costs’.

For hypothesis 3, we found support for the effect of diversification on efficient charity in both hypothesis 3a, the single-project condition, and hypothesis 3b, the multiple charities versus one condition. For hypothesis 3c, where the two provided charities had an equal efficiency, we found no support for an effect using a 0.005 alpha in the control condition in both studies 1 and 2, in line with the results of the original study. These results show support for the hypothesis that not all people allocate all funds to the more efficient charity and instead, on average, people tend to diversify their donations to allocate some money to the less efficient charity.

Unlike the original study, which analysed versions 1, 2 and 3 of hypothesis 3a in study 4 and reported that the responses between the three ‘did not differ in any meaningful way’, we found support for differences in responses between versions 1, 2 and 3 for almost all questions; the differences in responses, however, can be adequately explained by the differences in framing and efficiency in the three scenarios (i.e. charity A saves 50% more people than B in version 1, while charity A saves 25% more people than charity B in versions 2 and 3 though phrased differently; the average response is highest towards charity A in version 1 for all four questions asked).

Additionally, compared to an effect of *d* = 0.62 for single projects, the ‘several projects versus one’ sub-hypothesis instead had a larger effect of *d* = 1.18. One possible reason for the large effect size is that there is a difference in evaluability between the two types of scenarios; as the length of the scenarios in the several projects versus one sub-hypothesis is longer and thus harder to process, the lower evaluability of those scenarios may cause participants to become more biased towards the less cost-efficient charity. However, there may also be a methodological weakness in the questions used for hypothesis 3 as a whole, such that the responses of a few participants may impact the overall result. As the *t*‐test midpoint for hypotheses 3a and 3b is 100% allocation towards the more efficient charity, a few inattentive respondents who answer 50/50 allocation or give a random answer without parsing the question thoroughly, for example, may skew the results heavily away from the *t*‐test midpoint of ‘100% towards the more efficient charity’, thereby artificially inflating the effect size of any effect found. Nevertheless, we think that such a large effect size is indicative that the diversification effect does indeed exist, even if such a methodological weakness does cause an overestimation of the true effect size.

For hypothesis 4 (nationalism/ingroup effect), the results support the hypothesis that people tend to favour allocating more money to charities that offer aid to local communities over foreign ones. Unlike the original article which found that ‘the regions did not differ in allocation’, we found support for differences between regions in study 3; specifically, we found that the allocations in the India and Africa conditions were each different from the allocation in the China condition. But as we found support for this effect in each individual version with Cohen’s *d* values of 0.40 or above, and the magnitude of the differences between regions were very small (partial ω² = 0.00), we conclude that these differences are reflective of variations in perception by our US participants of these individual regions and that the ingroup effect as a whole can still be shown to be present.

### 4.2. Extensions

In hypothesis 6, contrary to our hypothesis that participants would be biased towards a charity that could provide them with an increased reputation, we instead observed the opposite effect: participants were instead biased towards the charity that kept their names anonymous, with an effect size of *d* = 0.52. Additionally, the qualitative feedback indicated that participants did indeed parse this item as being a manipulation of reputation (e.g. ‘I do not donate to get acknowledgement or accolades from anybody’, ‘if you give for recognition, that is the wrong reason’), making this effect unlikely to be the result of a confounding factor or a methodological error.

This result runs contrary to previous studies such as Andreoni & Petrie [45], which found that participants tend to prefer their donations be known to others instead of staying anonymous when given the choice, and Firmansyah & Pratma [46], which in a real-life sample of United States GoFundMe donors, found that only 33% of donors chose to remain anonymous. It also contrasts with studies such as Alpizar *et al*. [15], Peng *et al*. [47] and Vesely *et al*. [48], all of which found that participants whose actions were observable donated more, and Dwyer *et al*. [49], which in a real-life study found that generous spending was similar between a group asked to keep their spending private and a group asked to publicize them.

There are a few reasons for these results. One is that the results are due to signalling: when given a choice between anonymity and going public, people may choose anonymity over going public to signal that their motives are pure and that they do not wish to donate to increase their reputation. Another reason involves the ecological validity of this study: as the scenarios are merely hypothetical and do not involve real money, it may be that in actual scenarios, people may want to get their ‘money’s worth’ back from donations and ‘buy’ some reputation using the money that they donated, and signalling becomes less important in priority. We consider this a promising direction for future research.

Hypothesis 7, which found that participants were biased towards a charity where overhead is paid for by another donor even when cost-effectiveness is kept constant, acts as a conceptual replication of Gneezy *et al*. [16]. In contrast to the between-subject design that they used in their study, our extension shows that this effect occurs in a within-subject context as well. When participants are given details of both the control and the overhead conditions, thereby enabling them to directly compare the two—and, most importantly, allow them to realize that the charities in both conditions actually have an equal cost-effectiveness (e.g. one participant responded that ‘mathematically these [charities] sound the same’)—a bias towards the charity with the externally funded overhead is still present, meaning that the presence of such a bias is not due solely to an evaluability confound.

### 4.3. Limitations

#### 4.3.1. Hypothesis 5 (forced charity)

Hypothesis 5, the ‘forced-charity/government-taxes’ condition, presented us with a challenge to interpret when we were constructing this study. The original book chapter reported the results of five versions of the same hypothesis, of which two were reported as having no bias against forced charity. But the original authors then proceeded to report that ‘[they] have some evidence for a labile preference for voluntary mechanisms’ and concluded as such; thus, in our replication, we decided to conclude a successful replication of hypothesis 5 if we found support for the bias overall across the five versions, identical to the other hypotheses.

However, the results of the study showed us that there may have been a methodological error in the construction of the original data analysis for this hypothesis. Specifically, although we found support for a bias against forced charity in all five versions of hypothesis 5 when following the original study’s methods, *post hoc* comparisons showed that across all of these five versions, the responses to the question asking the participant ‘Which case provides more freedom of choice?’ were different from that of each of the other three questions. This can also be visually observed to be the case in electronic supplementary material, figure S2, where participant responses all clearly trend towards the ‘voluntary’ side of that question, in particular, across all five versions. This differs from the findings of the original book chapter, which reported that ‘answers to [all] the questions were highly correlated’.

Because all the questions for hypothesis 5 were designed such that one case involves forced charity and the other involves voluntary donations, it follows that if the manipulation worked, participants should be prompted to respond that the voluntary donation option would provide more freedom of choice than the forced charity option by definition, as the whole point of the manipulation is to see whether or not there is a bias between a forced option and a voluntary option in charitable giving. Therefore, we think that this specific question fails to provide insight as to which option the participant may be biased for or against, and does not do anything more than perhaps act as a comprehension check, indicating that the participant understands the ‘voluntary donations’ option as being one that provides more freedom of choice.

With this rationale in mind, we therefore decided to run the data analysis for all five versions of hypothesis 5 again as a *post hoc* exploratory analysis, but this time only using the data obtained from the other three questions (namely ‘Which case would you favour if you had a choice?’, ‘Which case is more fair in distributing the cost and benefits?’, and ‘Which is more important in this scenario?’). We summarize the analyses in table 8, in which we found that contrary to what was found when following the methods of the original book chapter, we failed to find support for a bias against forced charity in versions 1, 2, 3 and 4, and only found support for such a bias in version 5 with an effect size of *d* = 0.32.

However, unlike versions 1 through 4, which dealt with health insurance and tariffs and of which the negative consequences thereof did not directly threaten lives, version 5 asked participants what they would do in an epidemic (‘A new epidemic disease threatens to infect 10% and kill 1% of the children in your nation…’). As our replication data collection took place in 2023, with the memory of the global COVID-19 pandemic and its effects still fresh, we hypothesize that the effect seen in favour of voluntary donations in this specific version can be attributed to medical populism in the United States that occurred in response to the pandemic instead [50,51].

One key attribute of medical populism as identified by Lasco [50] is the ‘simplifying [of] the pandemic by downplaying its impacts’. In this version of hypothesis 5, the death rate is specifically mentioned to be ‘1% of [all] children’ in their country if the hypothetical disease is left unchecked; and yet in the qualitative feedback section, a participant replied that they ‘don't think it’s fair to raise taxes on everyone, especially when a disease has such a low [...] death rate’. It is obvious that the death of 1% of all children in a country as populous as the USA cannot, at any rate, be considered ‘low’. Another key attribute is the ‘forging [of internal] divisions between the “people” and dangerous “others”’; the ‘others’ in this case ‘include powerful elites such as pharmaceutical companies’. In this version of hypothesis 5, the hypothetical situation presented is that ‘[the treatment] costs too much for any insurance company to cover it, including current government insurance’; and in response, one participant replied that ‘I don't believe that medicines are too expensive to make, only that big pharmaceutical companies are too greedy’.

The support found for a bias against forced charity in version 5 of this hypothesis thus, instead of being a reflection of a more general bias against forced charity, may in fact be a response to the specific stimuli of ‘epidemic disease’ and ‘your nation’ caused by a distrust in the government and other organizations with respect to scenarios involving public health emergencies, especially when taking into account the failure to find support in each of the other four versions. In short, the support found for hypothesis 5 might not have been caused by an actual bias against forced charity, but may instead have been caused by the confounds of both methodological error and the effects of an unrelated political phenomenon.

In spite of these limitations to hypothesis 5, as we found support for all five replication hypotheses when following our pre-registered methods following that of the target article, we must conclude that the replication of this hypothesis (and thus all five hypotheses as a whole) was successful; but based on these *post hoc* exploratory analyses, we urge caution when interpreting the results of the replication of hypothesis 5 due to this caveat. Further research is needed to clarify whether a bias against forced charity exists in the way described in the original article and to define its range of effect if it does.

#### 4.3.2. Adjusted alpha threshold

Multiple hypotheses were tested in our replication. Additionally, multiple analyses were used to test each replication hypothesis. Hypothesis 3 even had three sub-hypotheses, each tested with multiple different analyses. This naturally raises the question of the possibility of *p*-hacking due to the large number of analyses involved.

Standard methods for dealing with multiple analyses pointing towards the same hypothesis include setting a stricter alpha level, which would reduce the risk that any support found for the existence of any given effect be the result of a type I error. In our replication, we set the alpha level for all studies to 0.005. However, this caused our methodology to differ from that of the original article, which used 0.05 as their alpha level across all analyses; this necessarily caused our threshold for concluding support to be much more strict than that of the original article, even if this may have been a methodological oversight on the part of the original authors. However, we believe that such a modification strikes a balance between adhering to the methods of the original studies as closely as possible and reducing the risk of committing one or multiple type I errors in the replication process.

### 4.4. Directions for further research

#### 4.4.1. Range of biases included

The biases included in this study represent a small subset of possible impediments against effective charity; they do not provide a comprehensive overview of all possible manipulations that could affect how a potential donor gives to charity. For example, a specific manipulation not included within the scope of this article is unit asking [52],^2^ in which a manipulation of scope insensitivity is affected by asking how much a potential donor would be willing to donate to a single needy individual, before asking how much that same potential donor would donate to all needy individuals in the same category, thereby causing donors to become more scope consistent.

Moreover, reviews on (in)effective altruism as a whole, such as Caviola *et al*. [10], provide a broader overview of obstacles to effectiveness that can each be individually analysed, and frame some of the effects analysed in this study within a wider context. For example, they note that donors generally have a bias for ‘proximate beneficiaries over distant ones’, and frame ingroup bias as being a manifestation of this bias in terms of spatial distance; they then extend this bias over biological distance (i.e. humans over animals) and temporal distance (i.e. current generations over future generations) as well.

These further manipulations and reframings represent a broader scope of impediments against effective altruism that is beyond the scope of investigation of this replication and extension. We believe that our methodology, however, will be able to provide a baseline framework for future studies to model and adapt when investigating a wider range of potentially impactful effects in the literature.

#### 4.4.2. Between subject designs and ecological validity

All of our manipulations were run in a within-subject context; participants were given two charities that differed in a given aspect and were asked to compare and contrast between them. It may be possible that, when these same manipulations are instead separated and provided to different participants in a between-subject context, participants may then react in ways that they would previously have not due to the difference in evaluability between manipulations. Such an effect has been previously shown to exist by studies such as Caviola *et al*. [4], which showed that evaluability played a key effect in the interaction between cost-effectiveness and overhead ratio; participants who were presented with only one charity donated, on average, more money to the charity with a lower overhead but a lower overall cost-effectiveness, whereas participants who were presented with both charities donated more to the charity with a higher overhead but a higher overall cost-effectiveness.

It would not be surprising if many of the other biases evaluated in this article, or indeed other biases not included in this article’s scope, would also be affected by whether the manipulations were presented to the participant in a within- or between-subject context, which would limit the generalizability of our findings. In real life, potential donors would be likely to encounter scenarios where they are given information about a single charity to consider, so there is much value in investigating whether these heuristics may become more or less apparent in between-subject scenarios.

In a broader sense, the generalizability of our findings may also be limited by a lack of ecological validity; participant biases may also be further affected by other factors, such as if real charity names were used as stimuli instead of charities A and B, and display effects that may not be found using our model. However, we also believe that our methodology can easily be adapted by future research with minimal change needed to investigate between-subject manipulations and other different stimuli as well. New studies may also introduce actual donations with real stakes and compare those to hypothetical scenarios (e.g. [48]).

## 5. Conclusion

Many different mechanisms may drive a person’s decision to donate to a given charity. Some of these mechanisms may cause a person to donate with suboptimal cost-effectiveness due to the usage of certain heuristics during decision-making. In a very close replication of Baron & Szymanska [2], we found support for the effects of a preference for lower perceived waste, lower past costs, for the ingroup and for having some diversification between charities. We also found some indication for a preference against forced charity on cost-effective donations, with some caveats regarding validity. Additionally, we also demonstrated in an extension that participants were also affected by manipulations of reputation and external funding for overhead costs. Future investigation of biases affecting charitable giving should focus on investigating a wider range of possible manipulations, especially ones that are closer to real-life scenarios.

## Data Availability

We have provided all materials, data, and code at: https://osf.io/bep78/files/osfstorage. Electronic supplementary material is available online at https://osf.io/esy8w [53].

## Footnotes

^1^
We would like to thank our reviewer Jonathan Berman for their input on a previous version of this item regarding confounding factors in its construction.

^2^
We would like to thank our reviewer Amanda Geiser for bringing the work of Hsee *et al.* [52] and Caviola *et al*. [10] to our attention as possible future directions.

## References

[1] Bekkers R, Wiepking P. 2011 A literature review of empirical studies of philanthropy. Nonprofit Volunt. Sect. Q. **40**, 924–973. (10.1177/0899764010380927)

[2] Baron J, Szymanska E. 2011 Heuristics and biases in charity. In The science of giving: experimental approaches to the study of charity (eds DM Oppenheimer, CY Olivola), pp. 215–235. London, UK: Psychology Press. (10.4324/9780203865972-24)

[3] Hsee CK. 1996 The evaluability hypothesis: an explanation for preference reversals between joint and separate evaluations of alternatives. Organ. Behav. Hum. Decis. Process. **67**, 247–257. (10.1006/obhd.1996.0077)

[4] Caviola L, Faulmüller N, Everett J, Savulescu J, Kahane G. 2014 The evaluability bias in charitable giving: saving administration costs or saving lives? Judgm. Decis. Mak. **9**, 303–316. (10.1017/s1930297500006185) 25279024 PMC4179876

[5] Read D, Loewenstein G. 1995 Diversification bias: explaining the discrepancy in variety seeking between combined and separated choices. J. Exp. Psychol. **1**, 34–49. (10.1037/1076-898x.1.1.34)

[6] Fox CR, Ratner RK, Lieb DS. 2005 How subjective grouping of options influences choice and allocation: diversification bias and the phenomenon of partition dependence. J. Exp. Psychol. **134**, 538–551. (10.1037/0096-3445.134.4.538) 16316290

[7] Mullen B, Brown R, Smith C. 1992 Ingroup bias as a function of salience, relevance, and status: an integration. Eur. J. Soc. Psychol. **22**, 103–122. (10.1002/ejsp.2420220202)

[8] James TK, Zagefka H. 2017 The effects of group memberships of victims and perpetrators in humanly caused disasters on charitable donations to victims. J. Appl. Soc. Psychol. **47**, 446–458. (10.1111/jasp.12452)

[9] Burtch G, Ghose A, Wattal S. 2014 Cultural differences and geography as determinants of online prosocial lending. MIS Q. **38**, 773–794. (10.25300/misq/2014/38.3.07)

[10] Caviola L, Schubert S, Greene JD. 2021 The psychology of (in)effective altruism. Trends Cogn. Sci. **25**, 596–607. (10.1016/j.tics.2021.03.015) 33962844

[11] Butts MM, Lunt DC, Freling TL, Gabriel AS. 2019 Helping one or helping many? A theoretical integration and meta-analytic review of the compassion fade literature. Organ. Behav. Hum. Decis. Process. **151**, 16–33. (10.1016/j.obhdp.2018.12.006)

[12] Berman JZ, Barasch A, Levine EE, Small DA. 2018 Impediments to effective altruism: the role of subjective preferences in charitable giving. Psychol. Sci. **29**, 834–844. (10.1177/0956797617747648) 29659341

[13] Nosek BA *et al*. 2022 Replicability, robustness, and reproducibility in psychological science. Annu. Rev. Psychol. **73**, 719–748. (10.1146/annurev-psych-020821-114157) 34665669

[14] Bradley A, Lawrence C, Ferguson E. 2018 Does observability affect prosociality? Proc. R. Soc. B **285**, 20180116. (10.1098/rspb.2018.0116) PMC5897647 29593114

[15] Alpizar F, Carlsson F, Johansson-Stenman O. 2008 Anonymity, reciprocity, and conformity: evidence from voluntary contributions to a national park in Costa Rica. J. Public Econ. **92**, 1047–1060. (10.1016/j.jpubeco.2007.11.004)

[16] Gneezy U, Keenan EA, Gneezy A. 2014 Avoiding overhead aversion in charity. Science **346**, 632–635. (10.1126/science.1253932) 25359974

[17] Camerer CF *et al*. 2018 Evaluating the replicability of social science experiments in Nature and Science between 2010 and 2015. Nat. Hum. Behav. **2**, 637–644. (10.1038/s41562-018-0399-z) 31346273

[18] Espinosa R. 2024 Understanding biases and heuristics in charity donations. Peer Community Regist. Rep. 100775. (10.24072/pci.rr.100775)

[19] Chan M, Feldman G. 2025 . Factors impacting effective altruism: revisiting heuristics and biases in charity in a replication and extensions registered report of Baron and Szymanska 2011. Received stage 2 endorsement from Peer Community in Registered Reports. (10.24072/pci.rr.100775) PMC12092110 40400516

[20] Feldman G. 2023 Registered report stage 1 manuscript template. (10.17605/OSF.IO/YQXTP)

[21] Jané M *et al*. 2024 Guide to effect sizes and confidence intervals (10.17605/OSF.IO/D8C4G)

[22] Simonsohn U. 2015 Small telescopes: detectability and the evaluation of replication results. Psychol. Sci. **26**, 559–569. (10.1177/0956797614567341) 25800521

[23] Litman L, Robinson J, Abberbock T. 2017 TurkPrime.com: a versatile crowdsourcing data acquisition platform for the behavioral sciences. Behav. Res. Methods **49**, 433–442. (10.3758/s13428-016-0727-z) 27071389 PMC5405057

[24] LeBel EP, Vanpaemel W, Cheung I, Campbell L. 2019 A brief guide to evaluate replications. Meta Psychol. **3**, MP.2018.843. (10.15626/mp.2018.843)

[25] LeBel EP, McCarthy RJ, Earp BD, Elson M, Vanpaemel W. 2018 A unified framework to quantify the credibility of scientific findings. Adv. Methods Pract. Psychol. Sci. **1**, 389–402. (10.1177/2515245918787489)

[26] Posit team. 2022 RStudio: integrated development environment for R. Posit Software, PBC. See http://www.posit.co/.

[27] R Core Team. 2022 R: a language and environment for statistical computing. Vienna, Austria: R Foundation for Statistical Computing. See https://www.R-project.org.

[28] Ben-Shachar M, Lüdecke D, Makowski D. 2020 effectsize: estimation of effect size indices and standardized parameters. J. Open Source Softw. **5**, 2815. (10.21105/joss.02815)

[29] Wickham H, Miller E, Smith D. 2023 haven: import and export ‘SPSS’, ‘Stata’ and ’SAS’ files. R package version 2.5.2. See https://CRAN.R-project.org/package=haven.

[30] Revelle W. 2022 psych: procedures for personality and psychological research. R package version 2.2.9. See https://CRAN.R-project.org/package=psych.

[31] Makowski D, Lüdecke D, Patil I, Thériault R, Ben-Shachar MS, Wiernik BM. 2023 Automated results reporting as a practical tool to improve reproducibility and methodological best practices adoption. CRAN. See https://easystats.github.io/report/.

[32] Wickham H. 2007 Reshaping data with the reshape package. J. Stat. Softw. **21**, 1–20. (10.18637/jss.v021.i12)

[33] Barnier J. 2022 rmdformats: HTML output formats and templates for ‘rmarkdown’ documents. See https://CRAN.R-project.org/package=rmdformats.

[34] Kassambara A. 2023 rstatix: pipe-friendly framework for basic statistical tests. R package version 0.7.2. See https://CRAN.R-project.org/package=rstatix.

[35] Patil I. 2021 statsExpressions: R package for tidy dataframes and expressions with statistical details. J. Open Source Softw. **6**, 3236. (10.21105/joss.03236)

[36] Wickham H *et al*. 2019 Welcome to the tidyverse. J. Open Source Softw. **4**, 1686. (10.21105/joss.01686)

[37] Singmann H, Bolker B, Westfall J, Aust F, Ben-Shachar M. 2023 afex: analysis of factorial experiments. R package version 1.2-1. See https://CRAN.R-project.org/package=afex.

[38] Wickham H, François R, Henry L, Müller K, Vaughan D. 2023 dplyr: a grammar of data manipulation. R package version 1.1.0. See https://CRAN.R-project.org/package=dplyr.

[39] Wickham H, Sievert C. 2016 ggplot2: elegant graphics for data analysis. Cham, Switzerland: Springer. (10.1007/978-3-319-24277-4)

[40] Patil I. 2021 Visualizations with statistical details: the ‘ggstatsplot’ approach. J. Open Source Softw. **6**, 3167. (10.21105/joss.03167)

[41] Larmarange J. 2022 labelled: manipulating labelled data. R package version 2.10.0. See https://CRAN.R-project.org/package=labelled.

[42] Pohlert T. 2022 PMCMRplus: calculate pairwise multiple comparisons of mean rank sums extended. R package version 1.9.6. See https://CRAN.R-project.org/package=PMCMRplus.

[43] Lüdecke D. 2022 sjlabelled: labelled data utility functions (version 1.2.0). (10.5281/zenodo.1249215)

[44] Knief U, Forstmeier W. 2021 Violating the normality assumption may be the lesser of two evils. Behav. Res. Methods **53**, 2576–2590. (10.3758/s13428-021-01587-5) 33963496 PMC8613103

[45] Andreoni J, Petrie R. 2004 Public goods experiments without confidentiality: a glimpse into fund-raising. J. Public Econ. **88**, 1605–1623. (10.1016/s0047-2727(03)00040-9)

[46] Firmansyah FM, Pratama AR. 2021 Anonymity in COVID-19 online donations: a cross-cultural analysis on fundraising platforms. In Advances in information and communication (ed. K Arai), pp. 34–47. Cham, Switzerland: Springer. (10.1007/978-3-030-73103-8_3)

[47] Peng Y, Li Y, Wei L. 2022 Positive sentiment and the donation amount: social norms in crowdfunding donations during the COVID-19 pandemic. Front. Psychol. **13**, 818510. (10.3389/fpsyg.2022.818510) 35265015 PMC8901185

[48] Vesely S, Klöckner CA, Carrus G, Chokrai P, Fritsche I, Masson T, Panno A, Tiberio L, Udall AM. 2022 Donations to renewable energy projects: the role of social norms and donor anonymity. Ecol. Econ. **193**, 107277. (10.1016/j.ecolecon.2021.107277)

[49] Dwyer RJ, Brady WJ, Anderson C, Dunn EW. 2023 Are people generous when the financial stakes are high? Psychol. Sci. **34**, 999–1006. (10.1177/09567976231184887) 37530643

[50] Lasco G. 2020 Medical populism and the COVID-19 pandemic. Glob. Public Health **15**, 1417–1429. (10.1080/17441692.2020.1807581) 32780635

[51] Lasco G, Curato N. 2019 Medical populism. Soc. Sci. Med. **221**, 1–8. (10.1016/j.socscimed.2018.12.006) 30553118

[52] Hsee CK, Zhang J, Lu ZY, Xu F. 2013 Unit asking. Psychol. Sci. **24**, 1801–1808. (10.1177/0956797613482947) 23907547

[53] Chan M, Feldman G. 2025 Supplementary material from: Factors impacting effective altruism: Revisiting heuristics and biases in charity in a Replication Registered Report of Baron and Szymanska (2011). Figshare. (10.6084/m9.figshare.c.7717993) PMC12092110 40400516

